# Multilevel interrogation of H3.3 reveals a primordial role in transcription regulation

**DOI:** 10.1186/s13072-023-00484-9

**Published:** 2023-04-07

**Authors:** Syed Nabeel-Shah, Jyoti Garg, Kanwal Ashraf, Renu Jeyapala, Hyunmin Lee, Alexandra Petrova, James D. Burns, Shuye Pu, Zhaolei Zhang, Jack F. Greenblatt, Ronald E. Pearlman, Jean-Philippe Lambert, Jeffrey Fillingham

**Affiliations:** 1 Department of Chemistry and Biology, Toronto Metropolitan University, 350 Victoria St, Toronto, M5B 2K3 Canada; 2 Donnelly Centre, University of Toronto, Toronto, M5S 3E1 Canada; 3 Department of Molecular Genetics, University of Toronto, Toronto, M5S 1A8 Canada; 4 Department of Biology, York University, 4700 Keele St, Toronto, M3J 1P3 Canada; 5 Department of Computer Science, University of Toronto, Toronto, M5S 1A8 Canada; 6 Department of Molecular Medicine, Cancer Research Center, Big Data Research Center, Université Laval, Quebec City, QC Canada; 7 CHU de Québec Research Center, CHUL, 2705 Laurier Boulevard, Quebec City, QC Canada

**Keywords:** H3.3, Histone variant, HIRA, CAF1, Asf1, NASP, RBBP4/7, Chromatin, Epigenetics, Functional proteomics, Tetrahymena

## Abstract

**Background:**

Eukaryotic cells can rapidly adjust their transcriptional profile in response to molecular needs. Such dynamic regulation is, in part, achieved through epigenetic modifications and selective incorporation of histone variants into chromatin. H3.3 is the ancestral H3 variant with key roles in regulating chromatin states and transcription. Although H3.3 has been well studied in metazoans, information regarding the assembly of H3.3 onto chromatin and its possible role in transcription regulation remain poorly documented outside of Opisthokonts.

**Results:**

We used the nuclear dimorphic ciliate protozoan, *Tetrahymena thermophila*, to investigate the dynamics of H3 variant function in evolutionarily divergent eukaryotes. Functional proteomics and immunofluorescence analyses of H3.1 and H3.3 revealed a highly conserved role for Nrp1 and Asf1 histone chaperones in nuclear influx of histones. Cac2, a putative subunit of H3.1 deposition complex CAF1, is not required for growth, whereas the expression of the putative ortholog of the H3.3-specific chaperone Hir1 is essential in *Tetrahymena*. Our results indicate that Cac2 and Hir1 have distinct localization patterns during different stages of the *Tetrahymena* life cycle and suggest that Cac2 might be dispensable for chromatin assembly. ChIP-seq experiments in growing *Tetrahymena* show H3.3 enrichment over the promoters, gene bodies, and transcription termination sites of highly transcribed genes. H3.3 knockout followed by RNA-seq reveals large-scale transcriptional alterations in functionally important genes.

**Conclusion:**

Our results provide an evolutionary perspective on H3.3’s conserved role in maintaining the transcriptional landscape of cells and on the emergence of specialized chromatin assembly pathways.

**Supplementary Information:**

The online version contains supplementary material available at 10.1186/s13072-023-00484-9.

## Introduction

The fundamental repeating unit of chromatin is the nucleosome which is composed of two copies each of the four core histones H2A, H2B, H3, and H4 around which about 147 bp of DNA is wrapped [1]. In most eukaryotes, two classes of histones have been described: (1) replicative or canonical histones, expressed only during the S phase of the cell cycle, which are assembled onto chromatin in a DNA replication-dependent (RD) manner, and (2) variant histones, which differ in their primary amino acid sequences, are expressed throughout the cell cycle, and can be deposited in a replication-independent (RI) fashion [2]. For example, in humans, H3.1 and H3.2 are two RD histones, whereas H3.3 is an RI variant histone.

The RI histone variants have an uneven distribution across the genome, carry specific posttranslational modifications (PTMs), and can affect gene expression by altering the chromatin state [3]. Human H3.3 differs from H3.1 and H3.2 at only five and four evolutionarily conserved amino acid residues, respectively [4]. H3.3 exhibits enrichment in gene bodies of actively transcribed genes, at promoter regions of both active and inactive genes, and at genic and intergenic regulatory regions in animal model systems [5]. Moreover, H3.3 accumulation over genes correlates with that of RNA polymerase II (RNAPII), indicating that H3.3 marks regions of active transcription [6, 7]. Human H3.3 has also been found to be enriched at telomeres, as well as at pericentric heterochromatin [8]. H3.3 has been linked to several human diseases, including cancer. For example, missense mutations at K27 and G34 of H3.3 have been observed in over 60% of pediatric high-grade gliomas, and mutations at K36 and G34 of H3.3 have been reported in over 90% of bone tumors [9–11].

Anti-silencing factor 1 (Asf1) and Nuclear autoantigenic sperm protein (NASP) are two generalized histone chaperones that function in the transport of newly synthesized histones H3(H3.3)/H4, as well as the buffering of excess histones [12–14]. RD and RI H3s are deposited onto chromatin by distinct chaperone complexes. Mammalian H3.1 and H3.2 are deposited by the heterotrimeric chromatin assembly complex 1 (CAF1), whereas H3.3 is deposited at transcriptionally active regions by the Histone Regulator A (HIRA) complex [15, 16]. The CAF1 complex consists of the three subunits in humans, i.e., p150, p60, and p48 (also RbAp48 or RBBP4) (Cac1, Cac2, and Cac3 in budding yeast, respectively). The p60 subunit of CAF1 consists largely of WD40 repeats, displays H3/H4 binding activity, and contains two B-domains that mediate CAF1 interaction with Asf1 [17, 18]. Budding yeast cells lacking *CAC1, CAC2,* and *CAC3* are viable; however, they are sensitive to a variety of DNA damaging agents [19]. The HIRA complex is composed of HIRA (Hir1, Hir2 in budding yeast), calcineurin-binding protein 1 (CABIN1), and Ubinuclein 1 (UBN1) [20, 21]. HIRA depletion causes severe defects during mouse embryonic development [22], and in budding yeast, mutations in *HIR* genes are known to display synthetic defects or lethality when combined with mutations in genes that encode the components of the transcription elongation factor (FACT) complex [23]. HIRA is also a WD40 repeat protein and contains Asf1-interacting B-domain sequences [24]. The prevailing view regarding histone deposition is that Asf1 escorts H3.1/H4 and H3.3/H4 dimers and transfers them to either the CAF1 or HIRA complex, respectively, which subsequently deposits them onto chromatin [15, 16]. H3.3 deposition at telomeres and pericentric heterochromatin regions takes place through a distinct chaperone complex DAXX–ATRX [8]. Neither DAXX nor ATRX is found in yeast, consistent with a recent evolutionary origin of these proteins.

The essentiality of H3.3 appears to be species dependent. For example, RD H3 can compensate for the loss of H3.3 in somatic tissues during *Drosophila melanogaster* development [25]. Similarly, H3.3 is not essential in *Caenorhabditis elegans* [26]. In contrast, a complete loss of H3.3 causes lethality in *Arabidopsis thaliana* [27]. *Saccharomyces cerevisiae* and *Schizosaccharomyces pombe* contain only one non-centromeric histone H3, which is closely related to H3.3 [28]. Both organisms, however, contain CAF1 and HIRA-like chaperones. In certain organisms, such as *Drosophila*, the deposition of H3.3 can occur via both RD and RI pathways [25]. Evolutionary studies have suggested that H3.3 is the ancestral form of H3.1/2 [4]. Even though H3.3 has been well characterized in metazoans, the dynamics of its incorporation, deposition complexes, and role(s) in transcription remained poorly examined in early branching eukaryotes.

*Tetrahymena thermophila* is a well-studied unicellular ciliate protozoan. *Tetrahymena* has two structurally and functionally distinct nuclei, a germline diploid micronucleus (MIC) and a somatic polyploid macronucleus (MAC), maintained within a single cytoplasm [29]. During vegetative growth, the MIC is transcriptionally silent and divides mitotically, whereas the MAC essentially controls all gene expression and divides amitotically [29]. Both nuclei are derived from the same zygotic nucleus during sexual reproduction (conjugation) [30]. During conjugation, two developing nuclei undergo meiosis and substantial chromatin alterations including DNA rearrangements and removal of ‘internally eliminated sequences’ (IES) [30–33]. Conjugation is initiated by mixing starved *Tetrahymena* cells of different mating types. In starved *Tetrahymena*, while the MAC remains transcriptionally active, the DNA replication and cell division are halted. At the onset of conjugation, the MIC enters meiosis, adopts a highly elongated shape referred to as the crescent, and becomes transcriptionally active. Transcription stops after the crescent stage, and MICs undergo two meiotic divisions, producing four identical haploid pronuclei. One of the progeny nuclei, the selected pronucleus, undergoes a prezygotic mitosis to produce two pronuclei, followed by the exchange of one pronucleus between the mating pairs and fusion to produce a zygotic nucleus. The zygotic nucleus undergoes two post-zygotic mitoses producing four nuclei, two of which become new MICs, while the remaining two develop as new MACs (NM, known as analgen). The parental MAC is degraded, and transcription is initiated from analgen. Owing to the nuclear dualism and separation of two chromatin states, *Tetrahymena* is an excellent experimental system to study chromatin-related processes and gene expression regulatory pathways [34, 35].

In addition to metazoans, H3 variants are also commonly found in ciliates. For example, 8 histone H3 variants have been detected in *Stylonychia lemnae*, although the functional relevance of these many histone genes has remained unknown [36, 37]. In *Euplotes crassus*, two distinct H3s have been identified that are differentially expressed at different stages of the life cycle, suggesting functional divergence of H3 in this organism [38]. In *Tetrahymena*, four non-centromeric histone H3 genes, *HHT1–HHT4,* have been identified [39]*. HHT1* and *HHT2* encode the same canonical RD H3 protein. Both canonical RD H3s are expressed during vegetative growth but repressed in starved cells. *HHT3* and *HHT4* encode H3.3-like variants. While *HHT3* (H3.3) is constitutively expressed in growing and starved cells [40, 41], *HHT4* (H3.4, not to be confused with testis-specific H3.4 found in animals) is very weakly expressed if at all [39]. A recent study has shown that the *HHT2* expression level peaks in S phase, while the *HHT3* level remains stable across the cell cycle in *Tetrahymena* [42]. Although, canonical H3s are strictly deposited in an RD manner, H3.3 and H3.4 can be deposited both by a transcription-associated RI pathway and inefficiently by an RD pathway [39]. Knockout (KO) studies have shown that neither the RD nor the RI H3s are essential for *Tetrahymena* growth [39]. *Tetrahymena* cells depleted of RD H3s grow more slowly than the wildtype unless either of the RI variants is overexpressed. H3.3 KO cells, although viable, exhibit developmental defects [39]. Furthermore, in H3.3 KO cells, H3.4 is upregulated suggesting functional redundancy among H3 variants in *Tetrahymena* [39]. Although the deposition pathways have been studied previously [39], the RD and RI deposition complexes and the role of *Tetrahymena* H3.3 in transcription regulation have not previously been investigated.

Here we utilized functional proteomics and genomics approaches to characterize the RD and RI H3 variants, their chaperones, and transcriptional regulatory role(s) of H3.3 in *Tetrahymena*. Our proteomics analyses identified highly conserved chaperones, including N1/N2(NASP)-related protein 1 (Nrp1) and Asf1^Tt^, as the major interaction partners for both H3 and H3.3. We found that Cac2 and Hir1 have distinct localization patterns during different stages of the *Tetrahymena* life cycle. Moreover, Cac2 appears to be dispensable for chromatin assembly, whereas Hir1 is an essential gene in *Tetrahymena*. Chromatin immunoprecipitation followed by high-throughput sequencing (ChIP-seq) experiments revealed a strong enrichment for H3.3 in the genic regions, particularly promoters, gene bodies, and near the transcription end sites of highly expressed genes during growth. Loss of H3.3 resulted in extensive remodeling of the transcriptome during vegetative growth. We suggest that H3.3 has an evolutionarily conserved role in maintaining the transcriptional landscape of cells and in fine-tuning the regulated expression of functionally important genes.

## Results

### Identification of *Tetrahymena* H3 and H3.3 interaction networks

**Fig. 1:**
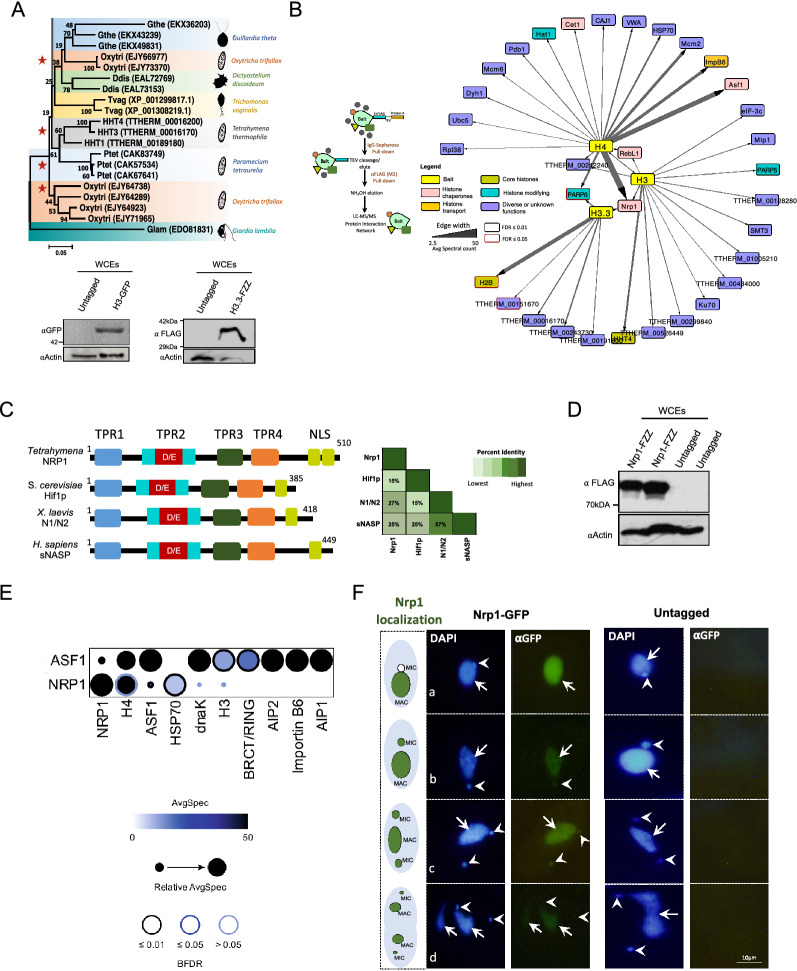
Identification of the H3 and H3.3 interactomes in *Tetrahymena*. **A** Top, Neighbor-joining phylogenetic analysis of RD and RI H3 proteins. Different species are highlighted in different colors. The numbers on the branches represent confidence values based on 1000 bootstrap replicates. Red stars indicate ciliates. Accession numbers are shown in brackets. Silhouettes adapted from http://phylopic.org/. Bottom, Western blotting analysis using whole cell lysates prepared from vegetative *Tetrahymena* cells expressing H3-GFP (H3 ∼ 15.43 kDa + GFP ∼ 27 kDa) and H3.3-FZZ (H3.3 ∼ 15.5 kDa + FZZ ∼ 18 kDa). The blots were probed with the indicated antibodies. **B** Left, Schematic representation of tandem affinity purification procedure. Right, Network representation of high-confidence (FDR ≤ 0.01) H3, H3.3, and H4 co-purifying proteins. See Additional file 1: Tables S1, S2 for complete AP-MS results. **C** Comparative domain analysis of *Tetrahymena* Nrp1 protein against *Homo sapiens*, *Xenopus laevis*, and *Saccharomyces cerevisiae* orthologs. Overall sequence identity among the orthologs is shown on the right. **D** Western blotting analysis using whole cell lysates prepared from growing *Tetrahymena* cells expressing Nrp1-FZZ (Nrp1∼ 59 kDa + FZZ ∼ 18 kDa). The blot was probed with the indicated antibodies. **E** Dot plot representation of high-confidence (FDR ≤ 0.01) Nrp1 and Asf1^Tt^ co-purifying proteins from vegetatively growing *Tetrahymena*. Inner circle color shows the average spectral count, the circle size indicates the relative prey abundance, and the circle outer edge is the SAINT FDR. See Additional file 1: Table S3 for complete AP-MS results for Nrp1. **F** Indirect immunofluorescence analysis of Nrp1-GFP in growing *Tetrahymena*. Nrp1 localization at different cell cycle stages is also indicated in the left panel. Untagged wildtype cells were used as a control. DAPI stained the nuclei, and the position of the MAC and MIC is indicated with arrows and arrowheads, respectively

We first analyzed the phylogenetic distribution of RD and RI histone H3s in early branching eukaryotes and observed that H3s clustered together based on species rather than variant types (Fig. 1A). The putative RD and RI H3 variants were well separated within each clade, suggesting a division between the RD and RI pathways in early branching eukaryotes (Fig. 1A). To explore RD and RI H3 variant dynamics in deep branching eukaryotes, we utilized *Tetrahymena* as a model system and identified H3 and H3.3 interaction partners that might assist in their deposition onto chromatin. Since *HHT4* is not expressed at appreciable levels in wildtype *Tetrahymena* cells, and *HHT3* is considered the major RI variant [39], we focused our studies on this variant. We engineered *Tetrahymena* cell lines stably expressing RD *HHT2* and RI *HHT3* with a C-terminal FZZ or GFP epitope tag from their endogenous MAC loci (Additional file 2: Fig. S1A, B). Both FZZ (3 × FLAG followed by two protein A moieties separated by a TEV cleavage site) and GFP epitope tags can be utilized in affinity purification as well as indirect immunofluorescence (IF) experiments. Western blotting assays in whole cell extracts (WCEs) prepared either from the epitope-tagged *HHT2* and *HHT3* cells or untagged wildtype *Tetrahymena* confirmed the successful expression of tagged H3 and H3.3 proteins (Fig. 1A). GFP or FZZ epitope-tagged histones have been previously shown to remain functionally competent and are deposited onto chromatin in *Tetrahymena* [39, 43]. Our IF analysis indicated that H3 localized in both MAC and MIC, whereas RI variant H3.3 was predominantly macronuclear, and only a faint signal was observed in the MIC in growing *Tetrahymena* (Additional file 2: Fig. S1C) [39, 44]. These results are consistent with previous studies showing that *Tetrahymena* H3.3 inefficiently enters the RD chromatin assembly pathway in MICs [39].

We used our established affinity purification (AP) coupled to tandem mass spectrometry (AP-MS) pipeline to identify the H3 and H3.3 interactomes (Fig. 1B) [45, 46]. The AP-MS data were curated using SAINTexpress which employs semiquantitative spectral counts for assigning a confidence value to individual protein–protein interactions [47]. Application of SAINTexpress to the AP-MS data for two biological replicates of H3 and H3.3 affinity purifications from growing *Tetrahymena* cells filtered against > 15 control experiments discovered several interaction partners that pass the cut-off confidence value (Bayesian FDR ≤ 0.01) (Fig. 1B; Additional file 1: Table S1, S2). Our analysis revealed that H3 co-purifies with 14 significant interacting partners. Notably, among these high-confidence H3 interaction partners were H4, histone chaperones Nrp1 and RebL1, DNA double-stranded break (DSB) repair protein Ku70, and Poly [ADP-Ribose] Polymerase 5 (PARP5) (FDR ≤ 0.01) (Fig. 1B). Application of SAINTexpress to the H3.3 AP-MS data revealed Nrp1 as a high-confidence interaction partner, in addition to two functionally uncharacterized metabolic proteins (FDR ≤ 0.01). Nrp1 is the ortholog of the human NASP protein, a key generalized H3(H3.3)/H4 chaperone that has been shown to function in multiple aspects of histone metabolism ranging from histone transport to buffering of excess histones [15].

To provide a more comprehensive view of the H3(H3.3)/H4 interactome, we included our recently published H4 AP-MS data in our analyses (Fig. 1B) [48]. We have previously reported that Asf1^Tt^ and Nrp1 likely function in the transport pathway of newly synthesized H3/H4 in *Tetrahymena* [46] and, consistent with this, Asf1^Tt^ was identified as a high-confidence interaction partner for H4. We also identified Asf1^Tt^ in the H3 and H3.3 interactomes; however, it did not pass our statistical cut-off (FDR ≤ 0.01). Additionally, RebL1 was identified as a shared interaction partner between H3 and H4 (FDR ≤ 0.01). RebL1 is the putative ortholog of the CAF1 subunit Cac3 in *Tetrahymena* [48]. The co-purification of RebL1 with both H3 and H4, but not with RI H3.3, is consistent with the role of CAF1 in RD assembly. Although the *Tetrahymena* genome appears to encode a single putative homolog of the RI chaperone HIRA, it was not detected in our H3.3 protein interaction data (see below). The co-purification of Nrp1 with all three examined histones, H3, H3.3, and H4, is consistent with the reported roles of NASP-family proteins as histone chaperones. To investigate its function(s) in *Tetrahymena* we further characterized Nrp1.

### Nrp1 localization in MIC is cell cycle-dependent during vegetative growth

Newly synthesized histones H3 (H3.3)/H4 are transferred through several protein complexes in the cytoplasm before their entry into the nucleus [13, 14]. Asf1 and NASP are two major chaperones that function in the supply of newly synthesized histones [15]. NASP-family proteins contain a highly conserved domain architecture with four TPR motifs (TPR1-4), where the second TPR is interrupted by acidic patches (SHNi-TPR) [49–52]. Multiple sequence alignment analysis revealed that the Nrp1 TPR motif architecture is conserved in *Tetrahymena*, where the interrupted TPR2 is flanked by TPR1 and TPR3/4 (Fig. 1C).

To characterize the histone supply chain-related chaperones in *Tetrahymena*, we engineered knock-in cell lines with tagged *NRP1-FZZ* and *NRP1-GFP* at the endogenous MAC site, then performed AP-MS using Nrp1-FZZ as a bait (Fig. 1D). Application of SAINTexpress to the AP-MS data identified two high-confidence Nrp1 interaction partners, Asf1^Tt^ and DnaK, the latter sharing similarity with the heat shock protein HSP70 (FDR ≤ 0.01; Fig. 1E; Additional file 1: Table S3). Analysis of the Asf1^Tt^-FZZ AP-MS data reciprocally identified Nrp1 as a high-confidence interaction partner [46] (Fig. 1E), consistent with their reported interaction across diverse eukaryotes. In *Tetrahymena*, two additional proteins, Aip1 (Asf1-interacting protein 1) and Aip2, have been suggested to function in the H3/H4 transport pathway due to their interaction with Asf1^Tt^ and Importinβ6 [46]. To further characterize their role in the Asf1^Tt^-Nrp1-Importinβ6 pathway, we engineered *Tetrahymena* cell lines stably expressing Aip1-FZZ from their native MAC loci and subjected them to our AP-MS pipeline (Additional file 2: Fig. S2A). Despite our numerous attempts, we could not successfully express Aip2-FZZ from its endogenous MAC locus. While Aip1 was successfully recovered in these experiments, no other interacting protein passed our statistical threshold (FDR ≤ 0.01) (Additional file 1: Table S4).

IF staining in growing *Tetrahymena* indicated that while Asf1 predominantly localized to the MIC and faintly to the MAC, Nrp1 primarily localized to the MAC during vegetative growth (Fig. 1F). During vegetative growth MAC and MIC divide by different mechanisms, i.e., amitosis and mitosis, respectively, and at different stages of the cell cycle [53]. The MIC S phase occurs immediately following its M phase, without any intervening G1 phase. For the MAC, there are well-defined G1, S, G2, and amitosis phases. During interphase, the MIC typically sits in a pocket-like recess in the MAC surface. At the initiation of mitosis, the MIC starts to move away from the MAC. Once the MIC finishes dividing, the MAC initiates its division, and the MIC enters S phase of the cell cycle due to the lack of a distinct G1 phase in *Tetrahymena*. Our IF analysis revealed that Nrp1 localized to the MIC when it started moving away from the MAC (Fig. 1F-b). The staining persisted until the end of cytokinesis, indicating that Nrp1 is present in the MIC during S phase (Fig. 1F-d). These results suggest that Nrp1 localization to the MIC is DNA replication dependent. Consistently, in starved cells when DNA replication is halted, Nrp1 was only observed in the MAC (Additional file 2: Fig. S2B). On the other hand, Aip1-FZZ predominantly localized in the cytoplasm of growing *Tetrahymena* cells. This diffused cytoplasmic signal (Additional file 2: Fig. S2C) suggests that Aip1 does not have nuclear functions. In contrast, the MAC-specific H2A variant Hv1-FZZ and the MIC-specific linker histone Mlh1-FZZ, which were used as controls in these experiments, exclusively localized to the MAC and MIC, respectively (Additional file 2: Fig. S2D), as reported previously [54], while untagged control *Tetrahymena* cells (Fig. 1F) showed no significant signal. These results suggest a conserved role for Nrp1 in the cell cycle-dependent supply of histones to the MIC and MAC, required for DNA replication during vegetative growth in *Tetrahymena*.

### Proteomic analysis of chromatin assembly complexes

**Fig. 2:**
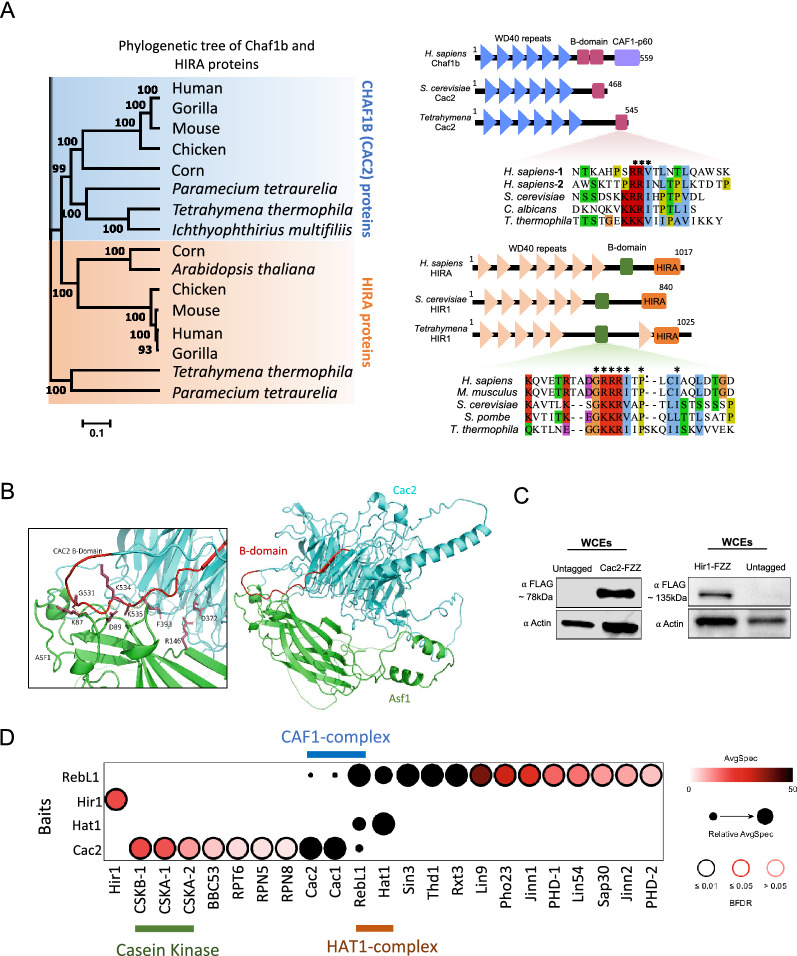
*Tetrahymena* Cac2 and Hir1 have highly conserved Asf1-interacting B-domain-like sequences. **A** Left, Neighbor-joining phylogenetic analysis of HIRA and Cac2 proteins. Different subfamilies are highlighted in different colors. The numbers on the branches represent confidence values based on 1000 bootstrap replicates. Right, Comparative domain analysis of *Tetrahymena* Cac2^Tt^ and Hir1^Tt^ proteins against *H. sapiens*, and *S. cerevisiae* orthologs. Highly conserved B-domain sequences are shown as multiple sequence alignments for both Cac2^Tt^ and Hir1^Tt^ proteins. **B** Visualization of the predicted binding interface between TTHERM_00219420 (Cac2) and TTHERM_00442300 (Asf1). Cac2^Tt^ is colored in cyan; Asf1^Tt^ is colored green. The B-domain of Cac2^Tt^ is highlighted in red. Labeled residues (K87-G531, D89-K534, R146-D372) are predicted to form polar intermolecular contacts between Asf1^Tt^ and Cac2^Tt^ within 3 Å, as well as an intramolecular π interaction (F393-K535) involving a Lysine residue within the B-domain of Cac2^Tt^ (T527–Y545). All interactions are shown as dashed yellow lines. **C** Western blotting analysis using whole cell lysates prepared from growing *Tetrahymena* cells expressing Cac2^Tt^ -FZZ (left; Cac2 ∼ 63 kDa + FZZ ∼ 18 kDa) and Hir1^Tt^-FZZ (right; Hir1 ∼ 117 kDa + FZZ ∼ 18 kDa). The blots were probed with the indicated antibodies. **D** Dot plot representation of the interaction partners identified with Cac2^Tt^, Hir1^Tt^, Hat1^Tt^, and RebL1 in growing *Tetrahymena* cells. Inner circle color shows the average spectral count, the circle size indicates the relative prey abundance, and the circle outer edge is the SAINT FDR. See Additional file 1: Tables S5, S6, and S8 for complete AP-MS data

In mammals and budding yeast, HIRA and Cac2 have been shown to interact with Asf1 (reviewed in Ref. [15]). Considering that neither of these proteins co-purified with Asf1^Tt^, nor did we identify any putative HIRA homologs as an H3.3 interaction partner (Fig. 1B and E), we aimed to identify and characterize these complexes in *Tetrahymena*. We have recently identified putative CAF1 subunits Cac1^Tt^ and Cac2^Tt^ via AP-MS of RebL1 (Cac3), which itself co-purified with histones H3 and H4 (Fig. 1B) [48]. To identify any putative HIRA homologs, we searched the *Tetrahymena* genome using budding yeast Hir1 and found a single gene, TTHERM_00046490, that appeared to encode a HIRA-like protein (Hir1^Tt^). To further characterize the identified proteins, we performed phylogenetic analysis using protein sequences from diverse eukaryotic species and observed that HIRA and Cac2 form two distinct clusters on the phylogenetic tree (Fig. 2A). While Cac2^Tt^ clustered together with its putative orthologs, Hir1^Tt^ did not group together with other HIRA orthologs (Fig. 2A), suggesting that Hir1^Tt^ might be functionally divergent. HIRA and Cac2 are WD-40 repeat-containing ancient paralogs that form β-propeller structures to provide a scaffold for mediating protein–protein interactions [20, 55]. Our sequence and structural analysis revealed that both Hir1^Tt^ and Cac2^Tt^ contain WD-40 repeats (Fig. 2A). Analysis of publicly available microarray data [56] revealed remarkable similarity in the expression profiles of Asf1^Tt^, Hir1^Tt^, and Cac2^Tt^ during *Tetrahymena* growth and development (Additional file 2: Figure S3), suggesting functional links among these proteins. Multiple sequence alignment analysis revealed the presence of a highly conserved Asf1-interacting B-domain in both Hir1^Tt^ and Cac2^Tt^ (Fig. 2A). We utilized the machine learning-based software AlphaFold2 to predict the structures of *Tetrahymena* Asf1^Tt^, Hir1^Tt^ and Cac2^Tt^ de novo [57]. The highly conserved N-terminal region of Asf1^Tt^ (1–156 amino acids) was predicted to form β-sheets organized into an Ig-like fold (Additional file 2: Fig. S4A–D), as has been shown in other organisms [24, 58]. As expected for the WD-40 repeat proteins, both Cac2^Tt^ and Hir1^Tt^ were predicted to fold into β-propeller-like structures (Additional file 2: Figs. S5, S6). The putative B-domains of both proteins were found outside of the predicted β-propellers. We performed protein complex predictions to model the interaction surfaces for Asf1^Tt^-Cac2^Tt^ and Asf1^Tt^-Hir1^Tt^ complexes. Cac2^Tt^ was predicted to form a highly stable complex with Asf1^Tt^ through its B-domain residues (Fig. 2B; also see Additional file 2: Figs. S7 and S8). Cac2^Tt^ residues G531, K534, and D372 were predicted to be within 3 Å distance from the Asf1^Tt^ residues K87, D89, and R146 (Fig. 2B). In contrast to Cac2^Tt^, however, no significant intermolecular interactions were predicted between Hir1^Tt^ and Asf1^Tt^ (Additional file 2: Fig. S9A). Although the unstructured regions of Hir1^Tt^ had low predictive confidence (Additional file 2: Fig. S9B), these results suggest that Hir1^Tt^ might be functionally divergent from its counterparts in humans and budding yeast, consistent with our phylogenetic analysis.

To further characterize and examine the interaction profiles of these proteins, we engineered epitope-tagged *Tetrahymena* cell lines expressing Hir1^Tt^-FZZ and Cac2^Tt^-FZZ from their native chromosomal loci (Fig. 2C) and subjected them to AP-MS analysis (Additional file 1: Tables S5, S6). While we did not detect any significant interaction partners for Hir1^Tt^-FZZ under our experimental conditions (FDR ≤ 0.01) (Additional file 1: Table S5), Cac2^Tt^-FZZ recovered a number of proteins after application of SAINTexpress to the AP-MS data, including the other two putative CAF1 subunits, Cac1 and RebL1 (Fig. 2D; Additional file 1: Table S6). Additionally, three putative subunits of the Casein kinase II complex (CKII) co-purified as high-confidence Cac2^Tt^-FZZ interaction partners (FDR ≤ 0.01; Additional file 1: Table S6). The CKII complex is a conserved serine/threonine kinase which, in humans and yeast, is composed of two regulatory beta subunits (CSKB) and two catalytic alpha (CSKA) subunits [59]. Two of the identified proteins, TTHERM_01345800 and TTHERM_01000180, shared sequence similarities with the budding yeast CSKA1 and CSKA2 subunits, respectively, whereas TTHERM_00780530 appeared to be the single putative homolog of the two regulatory beta subunits (hereafter designated as CSKB1) (Additional file 1: Table S6). Although further studies are required to examine the catalytic activity and functional significance of the CKII-CAF1 interaction, our AP-MS analysis of cells with tagged CSKB1-FZZ knocked into the endogenous site identified a large number of proteins, suggesting that CKII clients include functionally diverse proteins in *Tetrahymena* (Additional file 1: Table S7).

Despite numerous attempts, we could not successfully express Cac1^Tt^-FZZ from its native MAC locus. We therefore employed our recently reported RebL1 AP-MS data in our analysis to provide a comprehensive view of the putative CAF1 complex in *Tetrahymena* [48]. RebL1 interacts with multiple chromatin/transcriptional regulatory complexes, including CAF1 subunits and the histone acetyl transferase Hat1 [48]. In humans, two distinct proteins, RBBP4 and RBBP7 (Hat2 and Cac3 in yeast, respectively), function as subunits of the Hat1 and CAF1 complexes, respectively. We generated epitope-tagged *Tetrahymena* cell lines expressing Hat1-FZZ from its native MAC locus (Additional file 2: Fig. S10A) and subjected them to our proteomics pipeline. Application of SAINTexpress identified RebL1 as the sole high-confidence Hat1-FZZ interaction partner (Fig. 2D; Additional file 1: Table S8). These results further supported our previous findings that a single ortholog of RBBP4 and RBBP7 functions as a subunit of both the CAF1 and HAT1 complexes in *Tetrahymena*.

### Cac2 is dispensable for growth in *Tetrahymena*

**Fig. 3:**
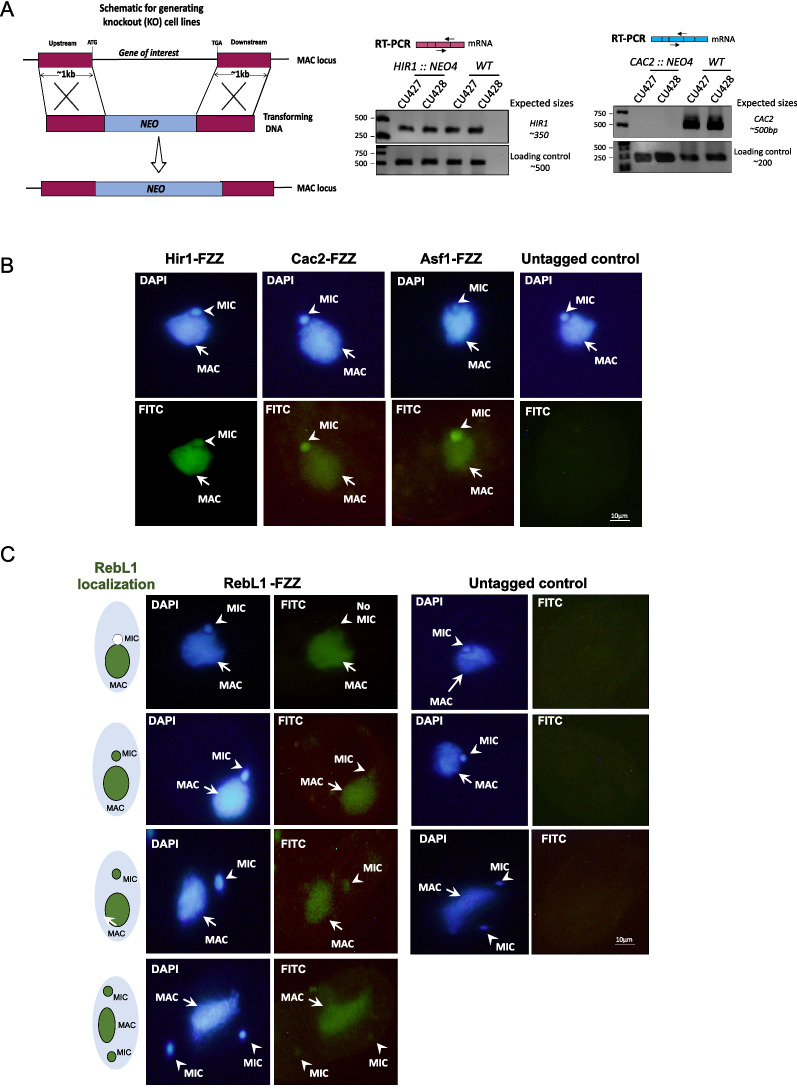
*Tetrahymena* Cac2 and Hir1 knockout analysis. **A** Left, Schematic representation of homologous recombination-mediated gene replacement strategy. The gene targeting vector carries a NEO drug marker which is flanked by 1 kb of DNA that shares sequence identity to upstream and downstream regions of the gene of interest. Right, RT-PCR analyses of *∆HIR1* and *∆CAC2* strains in comparison with wildtype *Tetrahymena* cells. The positions of the primers encompassing exon–exon junctions are indicated for both *∆HIR1* and *∆CAC2.* Bands were observed at the expected sizes. Primers specific to unrelated genes were used as loading controls. **B** Indirect immunofluorescence analysis of Hir1^Tt^-, Cac2^Tt^-, and Asf1^Tt^-FZZ in growing *Tetrahymena*. DAPI was used to stain the nuclei, and the positions of the MAC and MIC are indicated with arrows and arrowheads, respectively. **C** Indirect immunofluorescence analysis of RebL1-FZZ in dividing cells during *Tetrahymena* vegetative growth. DAPI was used to stain the nuclei, and the positions of the MAC and MIC are indicated with arrows and arrowheads, respectively. RebL1 localization at different cell cycle stages is also indicated as a cartoon in the left panel

To investigate the functions of these newly identified, putative RD and RI assembly complexes, we attempted to generate *CAC2*^*Tt*^ and *HIR1*^*Tt*^ knockout (KO) cell lines. We utilized homologous recombination-mediated gene replacement to replace the endogenous *CAC2*^*Tt*^ and *HIR1*^*Tt*^ loci with a drug resistance marker (*NEO4*) (Fig. 3A). PCR-based assays were used to verify the correct integration of the *NEO4* cassette into the targeted genomic loci (Additional file 2: Fig. S11). The *CAC2*^*Tt*^ and *HIR1*^*Tt*^ KO transformants were selected and screened under paromomycin resistance. While *CAC2*^*Tt*^ alleles were successfully replaced by the *NEO4* cassette, we could not obtain complete *HIR1*^*Tt*^ knockout strains (Fig. 3A) suggesting that it might be an essential gene. The *CAC2*^*Tt*^ KO cells, as well as *HIR1*^*Tt*^ knockdown (KD) cells, did not exhibit any growth defects, and cell proliferation was not significantly altered (data not shown). Although *CAC2*^*Tt*^ KO cells exhibited slightly enlarged MACs during vegetative growth, the difference was not statistically significant in comparison with the wildtype cells (*P*-value > 0.05) (Additional file 2: Fig. S12A). Furthermore, there was no significant difference in pairing efficiencies between the *CAC2*^*Tt*^ KO and wildtype cells, and conjugation proceeded normally (Additional file 2: Fig. S12B). Like wildtype mating pairs, *CAC2*^*Tt*^ KO and *HIR1*^*Tt*^ KD MICs underwent meiosis, forming a crescent, and 4 haploid products were observed in both the *CAC2*^*Tt*^ KO and *HIR1*^*Tt*^ KD cells (Additional file 2: Fig. S12B). Subsequent developmental events, including mitotic division, nuclear exchange, post-zygotic mitosis, analgen development, and degradation of the old MAC proceeded normally. The observation that loss of Cac2^Tt^ does not result in any major growth or developmental defect suggests that there might be additional chaperones and/or that there might be functional redundancy in chromatin assembly pathways in *Tetrahymena*.

### Cac2 and Hir1 have distinct localization patterns

To further characterize Cac2^Tt^ and Hir1^Tt^, we carried out indirect immunofluorescence experiments during vegetative growth. While Hir1^Tt^-FZZ localized to both MAC and MIC, Cac2^Tt^-FZZ predominantly localized to the MIC and faintly to the MAC (Fig. 3B). The localization profile of Cac2^Tt^ is similar to that of Asf1^Tt^, which also predominantly localized to the MIC during vegetative growth [46] (Fig. 3B). This observation also suggested that Cac2^Tt^ and Asf1^Tt^ might be functionally linked, consistent with our protein complex predictions. Unlike Cac2^Tt^, however, we found that both RebL1- and Hat1-FZZ localized predominantly to the MAC, and their localization to the MIC appeared cell cycle dependent (Fig. 3C, Additional file 2: Fig. S10B). These results suggest that a heterotrimeric CAF1 complex might not be present in the MICs of non-dividing *Tetrahymena* cells, and Cac2^Tt^ might have an additional MIC-specific function(s).

During starvation the MAC remains transcriptionally active; however, DNA replication is halted [29]. H3.3 has been found to localize exclusively to the MAC, whereas overexpressed H3 does not show any signal in either of the nuclei in starved cells [39]. Hir1^Tt^ did not exhibit any specific staining in starved cells as no signal was observed in either of the nuclei. Hir1^Tt^ is downregulated in starved *Tetrahymena* in comparison with growing cells, as revealed by microarray expression data (Additional file 2: Fig. S13A). This suggests that the observed diffuse pattern of staining might represent either non-specific signal in the absence of a strong target protein in starved *Tetrahymena* or Hir1^Tt^ is actively trafficked out of the nuclei (see below for conjugation IFs). Since H3.3 exclusively localizes to the MAC in starved cells (Additional file 2: Fig. S13B), this observation suggests that Hir1^Tt^ might not be required for transcription-associated RI deposition of H3.3 in the MAC. In contrast to Hir1^Tt^, Cac2^Tt^ localized exclusively to the MIC even though DNA replication was completely abolished, suggesting that the CAF1 complex is not actively trafficked out of MIC or destabilized. These results reinforce the idea that Cac2^Tt^ might have MIC-specific functions independent of its role in RD chromatin assembly. Moreover, consistent with cell cycle-dependent localization to the MIC, RebL1 showed only MAC localization in starved *Tetrahymena* (Additional file 2: Fig. S13C).

**Fig. 4:**
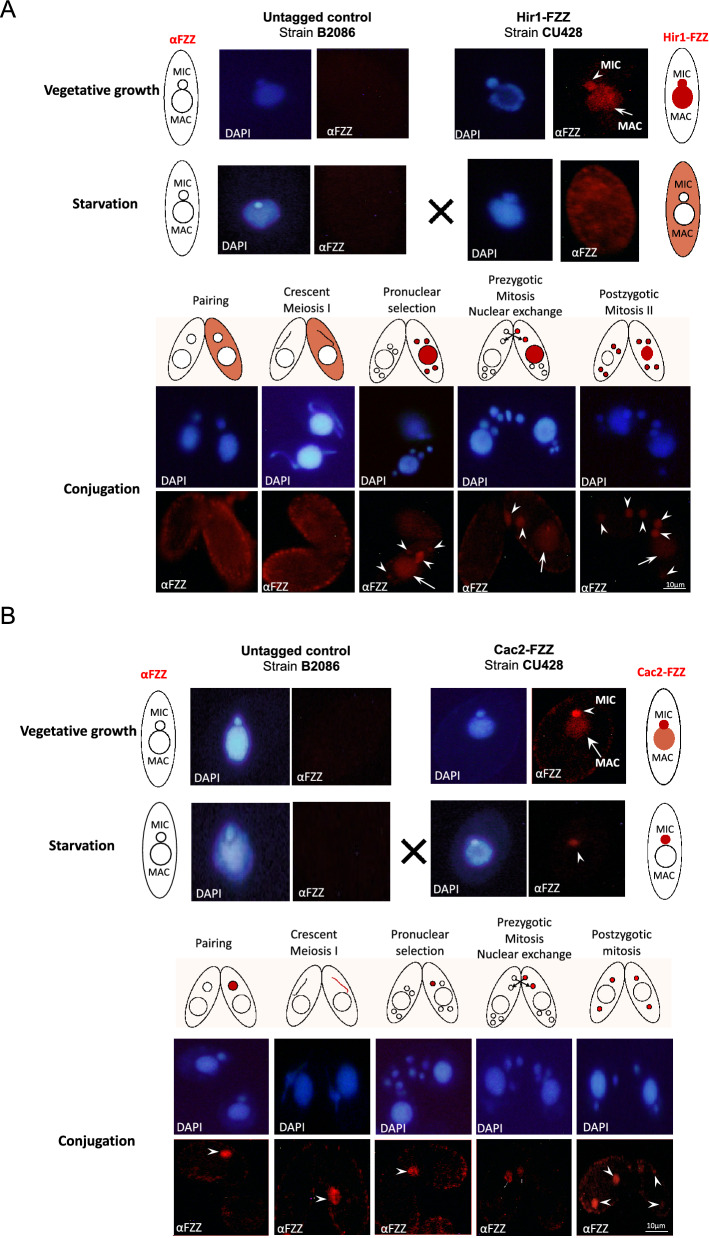
Cac2^Tt^ and Hir1^Tt^ show distinct localization during growth and development in *Tetrahymena*. **A** Hir^Tt^-FZZ localizes to both MAC and MIC during vegetative growth and exclusively to the cytoplasm during starvation. Hir^Tt^-FZZ cells were mated with untagged WT cells of different mating type. During conjugation, Hir1^Tt^-FZZ localizes to the four meiotic products and the parental MAC after the completion of meiosis. Hir1^Tt^-FZZ staining persisted in the parental MAC and the selected pronucleus only. **B** Cac2^Tt^-FZZ localizes predominantly to the MIC and faintly to the MAC during vegetative growth. Cac2^Tt^-FZZ signal was observed exclusively in the MIC during starvation. During conjugation, Cac2^Tt^-FZZ staining was observed in the crescent MIC as well as in the selected pronucleus. Note: Nuclear events are depicted above the images taken for conjugating cells during various developmental stages. DAPI was used to stain the nuclei. The signal observed in both mating types at the anlagen stage is due to the mixing of cellular contents between the pairing cells. CU428, mating type VII, and B2086, mating type II are the strain numbers of the strains obtained from the *Tetrahymena* Stock Center, Cornell University

In conjugating *Tetrahymena* cells, different nuclei have distinct patterns of replication, transcription, and recombination, and the localization profiles of the RD and RI H3 variants have been extensively documented in previous reports [29, 39]. In mating pairs of Hir1^Tt^-FZZ and WT cells, Hir1^Tt^-FZZ was not detected in meiotic nuclei, and signal appeared diffused throughout the cytoplasm, similar to untagged wildtype *Tetrahymena* (Fig. 4A). These results indicate that Hir1^Tt^ is not present in the crescent MICs, a stage when the MIC is transcriptionally active. After the completion of meiosis, Hir1^Tt^-FZZ signal appeared in the four meiotic products, in addition to the parental MAC (Fig. 4A). During *Tetrahymena* conjugation, one of the meiotic products is selected, whereas the remaining three nuclei are degraded. The selected nucleus undergoes prezygotic mitosis to produce two pronuclei, one of which is then exchanged between the mating pairs. Hir1^Tt^-FZZ signal was consistently observed in the MAC and in the selected pronucleus, whereas it disappeared from the non-selected nuclei (Fig. 4A). The signal persisted in the parental MAC and in the exchanged nuclei through post-zygotic mitosis-I and mitosis-II. In contrast to the Hir1^Tt^-FZZ, however, Cac2^Tt^-FZZ localized in the crescent nucleus, as well as in the selected pronuclei (Fig. 4B). No signal was observed in the MACs at any of the examined conjugation stages. The signal in the MICs persisted through the post-zygotic mitosis. It has been previously shown that the selected pronucleus undergoes chromatin remodeling required to produce mature gametes [60]. The localization profiles of Hir1^Tt^- and Cac2^Tt^-FZZ suggest a role for the putative RD and RI chaperones in the epigenetic reprogramming that occurs in pronuclei during *Tetrahymena* conjugation.

### H3.3 deposition profile in vegetative *Tetrahymena*

**Fig. 5:**
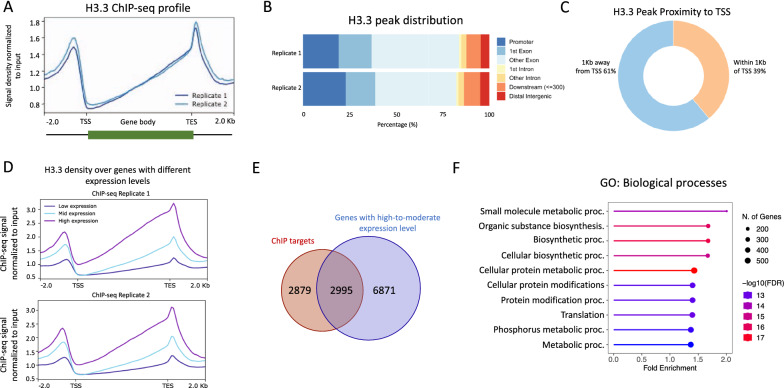
Genome-wide occupancy map of H3.3 in *Tetrahymena*. **A** Standardized metagene plot of H3.3 occupancy. **B** Bar plot depicting the H3.3 ChIP peak distribution with respect to annotated genomic features. **C** H3.3 ChIP peak distribution with respect to annotated TSS ± 1 kb. **D** Metagene plot showing the input normalized H3.3 ChIP-seq density over genes classified based on their expression levels during *Tetrahymena* growth. **E** Venn diagram showing the overlap of H3.3 bound genes with those genes that are classified as high-to-moderately expressed during *Tetrahymena* vegetative growth. **F** Lollipop plot representation of Gene Ontology (GO) terms significantly enriched in H3.3 ChIP-seq target genes (Q < 0.05). Also see Additional file 2: Fig. S14

In metazoans, H3.3 has been shown to be enriched in transcriptionally active chromatin domains, indicating a role in transcription regulation [5]. To investigate the genome-wide localization of H3.3, we utilized epitope-tagged *Tetrahymena* cell lines expressing H3.3-GFP from the native MAC loci and performed ChIP-seq experiments in vegetatively growing cells (Additional file 2: Fig. S14). Through peak calling in comparison with the input controls, we identified ∼ 5900 unique genes with reproducible peaks (FDR ≤ 0.05) (Additional file 1: Table S9). We found that H3.3 has the highest enrichment of reads over promoter regions, 1 kb upstream of transcription start sites (TSS), and transcription end sites (TES) (Fig. 5A). Peak distribution analysis indicated that the majority of the H3.3 peaks reside within gene bodies, primarily within exons (Fig. 5B). Furthermore, we found that ~ 39% of the peaks fall within 1 kb of the annotated TSS (Fig. 5C). These results are consistent with previous studies in mammalian cell lines showing the enrichment of H3.3 in promoter regions and gene bodies [5].

In mammalian cells, H3.3 has been reported to be enriched in highly expressed genes [6, 7]. To examine this possibility, we used publicly available RNA-seq data that has been used to rank *Tetrahymena* genes based on their expression levels during vegetative growth [61]. By plotting the H3.3 ChIP-seq signal, we observed that H3.3 occupancy positively correlates with gene expression levels, i.e., highly expressed genes exhibit substantially higher H3.3 density in comparison with the mid- and low-expression groups (Fig. 5D). About 51% of H3.3-target genes were highly to moderately expressed, whereas only 32% of the targets were classified as low- to no-expression during *Tetrahymena* growth (Fig. 5E). The remaining ~ 17% of H3.3 targets could not be classified into any expression group. These results suggest that H3.3 is deposited predominantly in highly transcribed genes in *Tetrahymena*, as previously observed in animals and plants [6, 7, 62].

Gene ontology (GO) enrichment analysis related to molecular functions and/or biological processes revealed that the H3.3-target genes were significantly enriched in pathways associated with highly transcribed genes involved in metabolic processes (Fig. 5F, Additional file 2: Fig. S15B). We further analyzed whether H3.3-occupied genes were enriched for any particular protein domains. We found that certain domains, including Myb-like DNA-binding, Ras, kinase, DnaJ, and domains commonly associated with metabolic enzymes, were significantly enriched (Additional file 2: Fig. S15C). We conclude that H3.3 exhibits a conserved chromatin occupancy profile and primarily targets highly expressed genes in *Tetrahymena*.

### Loss of H3.3 alters the transcriptional landscape of cells

**Fig. 6:**
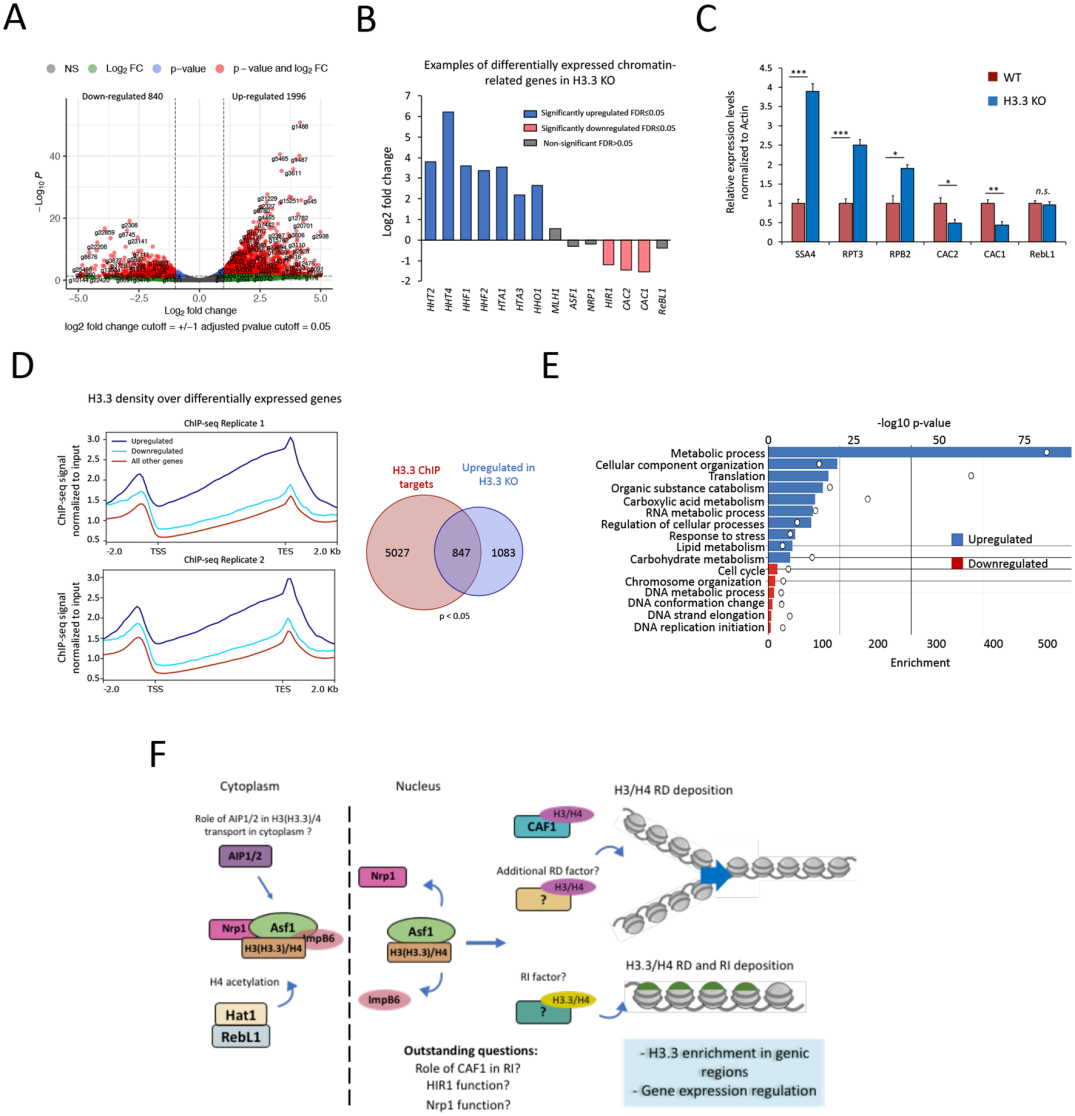
Loss of H3.3 remodels transcriptional landscape in *Tetrahymena*. **A** Volcano plot representation of genes differentially expressed in H3.3 knockout cells in comparison with the wildtype *Tetrahymena* cells. Each dot represents a single gene. Genes with FDR ≤ 0.05 were considered significant. Significant differential genes are shown as red dots with labels indicating the gene name. A legend is provided. NS: non-significant. **B** Bar plot showing the RNA-seq expression levels of selected genes in H3.3 KO cells. **C** Bar graphs showing RT-qPCR results to examine the differential expression of selected genes in H3.3 KO cells. The experiments were performed in biological triplicates, and p-values were calculated using the student’s *t*-test (∗∗∗*p* ≤ 0.001, ∗∗*p* ≤ 0.01, ∗*p* ≤ 0.05, n.s.: non-significant). Error bars represent standard error of mean (SEM). **D** Metagene plot showing the input normalized H3.3 ChIP-seq density over differentially expressed genes in H3.3 KO cells in comparison with unaffected genes (left). Venn diagram represents the overlap of significantly upregulated genes in H3.3 KO cells with H3.3 ChIP targets (right). *P*-value was calculated using the hypergeometric test. **E **GO enrichment analysis related to biological processes for differentially expressed genes in H3.3 KO cells. **F** Proposed model for H3 (H3.3)–H4 nuclear transport and chromatin assembly in *Tetrahymena*. The roles of Nrp1-Asf1 in H3/H4 transport and CAF1 in RD chromatin assembly appear conserved in *Tetrahymena,* whereas RI deposition, and identity of its cognate chaperone, requires further investigation

*Tetrahymena* cells lacking either the single RI variant or double knockouts (*ΔHHT3 ΔHHT4*) are viable for vegetative growth, and no major defects in global chromatin structure have been observed [39]. To further investigate H3.3’s role in maintaining the transcriptional landscape of cells, we utilized the H3.3 somatic KO *Tetrahymena* in which all macronuclear copies of the *HHT3* gene were replaced with the Neo drug resistance marker [39]. We performed RNA-seq in biological replicates using Poly A enriched RNA derived from *ΔHHT3* cells in parallel with parental wildtype *Tetrahymena*. Differential expression analysis identified 2836 genes that exhibited significant differential expression in *ΔHHT3* cells (Log2foldchange ≥ 1; adjusted *p*-value ≤ 0.05) (Additional file 1: Table S10). Among these differentially expressed genes, 1996 were significantly upregulated, whereas 840 were downregulated (Fig. 6A). Importantly, *HHT4,* which has been previously shown to be upregulated in *ΔHHT3* cells [39], was also identified as highly upregulated (> sixfold change) in our RNA-seq data (Fig. 6B). Additionally, certain histones, including H3, H4, H2A, and macronuclear linker histone Hho1, were also significantly upregulated. While the reasons for the observed upregulation of certain histones remain unclear, it might be a compensatory mechanism in response to the loss of H3.3 [39]. On the other hand, the transcript levels of micronuclear linker histone Mlh1, as well as the putative H3/H4 chaperones Asf1 and Nrp1, did not significantly change (Fig. 6B). It is also worth noting that the expression levels of Hir1^Tt^, Cac1^Tt^, and Cac2^Tt^ were also significantly reduced in H3.3 KO cells (Fig. 6B). We validated several of the H3.3 KO-dependent gene expression alterations, including those of Cac1, Cac2, Rpb2, Sas2, and Hsp90, using RT-qPCR experiments (Fig. 6C). RebL1 served as a negative control in these experiments.

When we correlated the gene expression changes with H3.3 occupancy, we observed that H3.3 was substantially more enriched in the upregulated genes in comparison with the downregulated genes or all other genes classified as ‘unaffected’ in *ΔHHT3 Tetrahymena* (Fig. 6D, left). Upregulated genes were found to have a significant overlap with H3.3 targets (*p*-value < 1.368e^−85^, hypergeometric test) (Fig. 6D, right). Furthermore, 42% of the upregulated genes were classified as ‘highly expressed’ in contrast to only 3% of downregulated genes (Additional file 2: Fig. S15A). These data are consistent with the above-described results indicating that H3.3 is deposited to highly transcribed genes and suggest that H3.3 might negatively regulate the expression of target genes.

GO enrichment analysis was performed to examine whether the identified differential genes were enriched for any particular biological processes and/or molecular functions. Our analysis revealed that the upregulated genes were significantly enriched for several important biological processes, including RNA-related processes, translation, and metabolic pathways (FDR < 0.05) (Fig. 6E). The downregulated genes, on the other hand, were significantly enriched for cell cycle regulation, DNA replication, and chromatin organization. Since H3.3 occupies highly expressed genes, and the majority of the affected genes in H3.3 KO cells were upregulated, these findings suggest that H3.3 might function to tightly regulate the expression of functionally important highly transcribed genes in *Tetrahymena*.

## Discussion

The dynamics of H3.3 have been well documented in metazoans [63]. However, the deposition complexes and H3.3 transcription-related roles have remained poorly studied outside of metazoans. We have previously reported that the core histones H2A, H2B, and H2A variant Hv1 (H2A.Z) are connected to a network of highly conserved chaperones and karyopherins in the ciliate *Tetrahymena* [43]. We have also shown that the linker histones, Mlh1 and Hho1, interact with functionally diverse proteins and lack any conserved chaperoning network in *Tetrahymena* [64]*.* Here, we utilized iterative proteomics, KO studies, and functional genomics approaches to examine the dynamics of the H3 variant in *Tetrahymena*.

Our finding that Asf1^Tt^ and Nrp1 interact with each other and co-purify with H3(H3.3)/H4 is consistent with a principal role for these proteins in the histone transport pathway. NASP is predominantly a nuclear protein that exhibits H3/H4-binding [65–67], and its expression is cell cycle regulated in mammalian cells [68]. Nrp1 localization to the MIC in a cell cycle-dependent manner suggests a role in the influx of newly synthesized histones required for DNA replication. A recent report has also indicated the cell cycle-dependent localization of Nrp1 to MICs and its role in H3 transport [69]. These authors also performed KO studies and showed that Nrp1 is required for gametic nuclei formation during conjugation [69]. Furthermore, Nrp1 has also been described to affect H3K56ac in *Tetrahymena* [70]. These studies are consistent with diverse roles of NASP-family proteins in H3/H4 metabolism and genome replication. Newly synthesized H4s are diacetylated at conserved residues K5 and K12 [71]. These deposition-related acetylation marks are installed by the Hat1/Hat2 complex [72]. Hat2 and Cac3 (RBBP7 and RBBP4 in humans, respectively) are homologous proteins that function as subunits of the Hat1 and CAF1 complexes, respectively. Consistent with our recent report [48], our AP-MS data indicate that RebL1 is the sole ortholog of Hat2 and Cac3 and functions as a subunit of both the putative Hat1 and CAF1 complexes in *Tetrahymena*. In addition to NASP and Asf1, certain cytoplasmic proteins have also been implicated in the regulation of H3/H4 transport. For example, in human cells, Codanin-1 has been shown to regulate the histone supply chain by sequestering Asf1 in the cytoplasm [73]. Aip1 and Aip2 are two protist-specific uncharacterized proteins that do not appear to contain any recognizable domains. Considering their interaction with H3/H4 and chaperones and cytoplasmic localization, an interesting hypothesis is that Aip1/2 function in the cytoplasm to regulate histone transport to the MIC and MAC in *Tetrahymena*. KO studies combined with monitoring H3/H4 transport to nuclei will be useful to test this hypothesis.

Although both putative RD and RI chromatin assembly factors Cac2^Tt^ and Hir1^Tt^ contain Asf1-interacting B-domain-like sequences, AP-MS analyses did not identify physical interactions with Asf1^Tt^. While we cannot rule out that Asf1^Tt^ interaction with Cac2^Tt^ and Hir1^Tt^ might be too transient and/or unstable to be identified under our stringent experimental conditions, another possibility is the divergent nature of these proteins in *Tetrahymena*. This possibility is consistent with our protein complex prediction studies which did not show any intermolecular interactions between Hir1^Tt^ and Asf1^Tt^. Although the observed robust signal on Western blots suggests that Hir1^Tt^-FZZ replaced most, if not all, endogenous copies of Hir1^Tt^, additional experiments to ensure the complete replacement will assist in further establishing the functionality of epitope-tagged Hir1^Tt^-FZZ, since HIR1 appears to be an essential gene. We have previously shown that expression of Asf1^Tt^ is essential in *Tetrahymena* [46]. Although the similarities in expression and localization profiles of Asf1^Tt^, PCNA1^Tt^ (Proliferating Cell Nuclear Antigen) [74], a key factor required for DNA replication, and Cac2^Tt^ are suggestive of a functional link among these proteins, we found that *CAC2*^*Tt*^ is not an essential gene in *Tetrahymena*. This suggests that there might be functional redundancy in chromatin assembly complexes in *Tetrahymena*. Expression of Hir1^Tt^, on the other hand, was found to be essential for vegetative growth in *Tetrahymena*. In mammals, HIRA forms a complex with two other proteins, CABIN1 and UBN1 [20, 21]. The *Tetrahymena* genome appears to encode at least two divergent putative orthologs of UBN1, TTHERM_00113110 and TTHERM_00335810 [20]. Although neither of these proteins co-purified with Hir1^Tt^, our somatic KO experiments suggest that at least one of these putative UBN1s, TTHERM_00113110, is also an essential gene in *Tetrahymena* (data not shown). Further studies are needed to explore the interactions and any possible functional links among these proteins. Moreover, a germline KO analysis will be helpful to further understand the possible functional relevance of Hir1^Tt^ in H3.3 deposition.

A key finding of our work is the distinct localization profiles of Cac2^Tt^ and Hir1^Tt^. Upon starvation, DNA replication is completely halted in both MIC and MAC [29]. Even though H3.3 can be deposited via both RD and RI pathways and is found in both nuclei during growth, it is only localized to the MAC in starved *Tetrahymena* [39]. This is consistent with a transcription-associated RI deposition pathway, since the MAC remains transcriptionally active in starved cells. Neither Cac2^Tt^ nor Hir1^Tt^ showed any staining in the MACs of starved cells. While Hir1^Tt^ is downregulated during starvation, and IF staining appeared non-specifically diffused throughout the cytoplasm, Cac2^Tt^ was exclusively found in the MIC. This suggests the possibility of another, yet unidentified, chromatin assembly pathway that deposits H3.3 in the MACs of starved *Tetrahymena*. The mechanism of H3.3 RI deposition remains unknown in *Tetrahymena*, since we have not been able to demonstrate an interaction between Hir1^Tt^ and H3.3. Although it is possible that the Hir1^Tt^ and H3.3 interaction might be too weak to be detected under our stringent experimental conditions, another possibility is that *Tetrahymena* Hir1^Tt^ is functionally divergent and does not take a major part in RI deposition. Another scenario is that the pool of free H3.3 in *Tetrahymena* is small due to quick assembly into the chromatin which may render H3.3-Hir1^Tt^ interaction below the detection limit of our experimental system. Further experiments will be needed to fully explore the role(s) of Hir1^Tt^ in histone metabolism. For example, overexpression of epitope-tagged H3.3 (under the MTT1 promoter) may reveal its interaction with Hir1^Tt^. Nrp1 and RebL1 could be potential candidates for roles in the RI pathway in *Tetrahymena*. Although, RebL1 localization to the dividing MIC indicates its critical role in RD assembly, this suggestion is consistent with RebL1 and Nrp1 MAC-specific localization in starved *Tetrahymena* and their known role(s) as histone chaperones in humans and budding yeast [15]. From an evolutionary perspective, it is conceivable that multiple, functionally redundant, chromatin assembly proteins existed in the last eukaryotic common ancestor, and over the course of evolution, more specialized chromatin assembly complexes and pathways emerged, presumably through neo/sub functionalization. Consistent with this notion, two Asf1 proteins in an evolutionary basal organism, *Trypanosoma brucei*, have been shown to have distinct subcellular localizations and functions [75].

We found that Cac2 and RebL1, the two examined CAF1 subunits, show distinct localization profiles during *Tetrahymena* growth. Considering that RebL1 localizes to the MAC in non-dividing cells, and Cac2^Tt^ predominantly localizes to the MIC, an attractive hypothesis is that the two *Tetrahymena* nuclei contain two structurally/compositionally distinct, stable, CAF1 complexes, i.e., a MIC-specific complex composed of Cac1^Tt^–Cac2^Tt^ and a MAC-specific heterotrimeric CAF1 complex composed of Cac1^Tt^, Cac2^Tt^, and RebL1. Consistent with this idea, Cac3 has been shown to be dispensable for CAF1-mediated chromatin assembly in budding yeast [76]. It is also worth noting here that CKII subunits were identified only with Cac2^Tt^ and not with RebL1. Although Cac2^Tt^ was not recovered in our reciprocal purifications and further studies are needed to examine the significance of this interaction, our reported analysis suggests that both Cac1^Tt^ and Cac2^Tt^ harbor conserved CKII phosphorylation sites (Additional file 2: Fig. S16). This suggests that CKII interaction might be in the context of a dimeric Cac1^Tt^–Cac2^Tt^ complex. RebL1 localizes to the MIC during S phase and this could represent RebL1’s function(s) in the acetylation of newly synthesized H4 as a HAT complex subunit and/or in RD chromatin assembly as a CAF1 subunit. Since CAF1 has a fundamental role in DNA replication-associated chromatin assembly, it will be important to further examine its MIC- and MAC-specific role(s).

The exclusive MIC localization of Cac2^Tt^ in starved cells also suggests that it might have functions independent of its role in RD chromatin assembly. Because the MIC enters conjugation with the 4C amount of DNA [77], Cac2^Tt^ localization exclusively to the MICs in starved cells suggests a function in preparing the cells for the onset of conjugation. In conjugating *Tetrahymena*, H3 localizes in the meiotic MICs at the crescent stage but not in the transcribing MACs of the same cells [39]. This H3 deposition is related to DNA repair synthesis associated with meiotic homologous recombination that occurs at this stage, and not with the genome duplication [78]. It is therefore possible that CAF1 might have a function(s) in DNA double-strand break (DSB) repair, since Cac2^Tt^ localized to the meiotic MICs. Consistent with this, in budding yeast CAF1 and Asf1 have been shown to function in DSB repair [79]. After the introduction of DSBs in pronuclei, ‘the selected pronucleus’ undergoes massive chromatin remodeling prior to fertilization, resulting in the formation of euchromatin [60]. It was also shown that Asf1^Tt^ appears in the selected pronucleus in response to DSBs [60]. We found that both Hir1^Tt^ and Cac2^Tt^ also localize to the selected pronucleus. This observation is consistent with a role in DSB repair, chromatin remodeling, and subsequent formation of euchromatin, possibly involving the H3.3 deposition that occurs in the selected pronucleus. Although the role of Hir1^Tt^ in RI assembly remains enigmatic, our findings suggest that newly synthesized histones H3(H3.3)/H4 are transported to the nuclei via an Asf1-Nrp1 pathway and are deposited onto chromatin through chaperone complexes that might be functionally redundant (Fig. 6F).

In mammalian cells, H3.3 is deposited in distinct chromatin regions including telomeric heterochromatin, gene bodies, enhancers, and promoters [5]. H3.3 deposition at highly expressed genes appears to be conserved in plants, *Drosophila* and mammals [5, 25, 62]. *Tetrahymena* H3.3 exhibited enrichment over promoters, gene bodies, and transcription termination regions of highly transcribed genes. In *Drosophila* and human cells, enrichment of H3.3 in the gene body and after the TES has been found to be correlated with transcriptional activity [5, 25]. Although the exact chaperone(s) and mechanism(s) of its deposition remain unknown, the overall binding profile of *Tetrahymena* H3.3 is consistent with a role in transcription regulation and euchromatin formation. In mice, loss of H3.3 genes results in developmental retardation and early embryonic lethality [80]. Although H3.3 is not essential for *Tetrahymena* growth, possibly due to compensation by other H3 variants, its loss results in severe developmental defects [39]. In *Drosophila*, H3.3-deficient animals exhibit large-scale gene expression alterations, with genes being both up- and downregulated [25]. We found that the loss of H3.3 results in wide-spread transcriptional defects in growing *Tetrahymena*. The observations that H3.3 occupies highly expressed genes and that such genes are upregulated upon H3.3 depletion supports the idea that H3.3 is required to ensure the regulated expression of highly expressed genes in *Tetrahymena*. From a mechanistic point of view, it remains possible that the observed upregulation of target genes might be related to reduced nucleosome density in highly transcribed genes in H3.3 KO cells. This may lead to accumulation of cryptic transcripts, as has been previously observed in *Arabidopsis* [27]. Further studies are needed to provide details of the exact mechanism.

In summary, our study has elucidated the proteomics, as well as functional aspects, of the RD and RI H3 variants in a ciliate and, more broadly, has extended current understanding of the evolutionarily conserved role of H3.3 in transcription.

## Material and methods

### Cell strains

*Tetrahymena* wild type strains CU428 [Mpr/Mpr (VII, mp-s)] and B2086 [Mpr+/Mpr+ (II, mp-s)] of inbreeding line B, as well as *∆HHT3* (Stock ID: SD01318) cells were obtained from the *Tetrahymena* Stock Center, Cornell University, Ithaca N.Y. (http://tetrahymena.vet.cornell.edu/). Cells cultured were maintained axenically at 30 °C in 1 × SPP media as previously described [46].

### Macronuclear gene replacement

Epitope tagging vectors for *Tetrahymena* genes were constructed as previously described [46]. We used wildtype *T. thermophila* genomic DNA as template and primers as indicated in Additional file 1: Table S11 to amplify two separate ~ 1 kb fragments upstream and downstream of the predicted stop codons of genes of interest. The PCR products were digested with KpnI/XhoI (upstream product) and NotI/SacI (downstream product). The digested products were cloned into the tagging vectors (pBKS-FZZ and pBKS-GFP), provided by Dr. Kathleen Collins (University of California, Berkeley, CA). The final plasmid was linearized by digesting it with KpnI and SacI prior to transformation. One micrometer gold particles (60 mg/ml; Bio-Rad) were coated with at least 5 μg of the DNA. The gold particles were introduced into the *T. thermophila* MAC using biolistic transformation with a PDS-1000/He Biolistic particle delivery system (Bio-Rad). The transformants were selected using paromomycin (60 μg/ml). MAC homozygosity was achieved by growing the cells in increasing concentrations of paromomycin to a final concentration of 1 mg/ml.

For knockout (KO) experiments, essentially the same strategy as described above was used except that the two separate DNA fragments (∼ 1 kb) upstream and downstream of the gene of interest were cloned into the gene targeting vector p4T2-1, which contained the Neo2 drug resistance gene. RT-PCR followed by agarose gel electrophoresis was used to assess the correct integration of the drug resistance cassette and homozygosity. Primers used are listed in Additional file 1: Table S11.

### Experimental design for mass spectrometry

At least two independent biological replicates of each, as well as negative controls, were processed in each batch of samples. As a negative control, we used wildtype *Tetrahymena* cells without tagged bait (i.e., empty cells). To reduce carry-over, we performed extensive washes between samples (see details for each instrument type). Additionally, the order of sample acquisition on the mass spectrometer was reversed for the second replicate to avoid systematic bias. On the LTQ mass spectrometer, a freshly made column was used for each sample, as previously described [46].

### Affinity purification and mass spectrometry sample preparation

Affinity purification was carried out essentially as described [45, 46]. *Tetrahymena* cells were grown to mid-log phase in ~ 500 ml of 1 × SPP to a final concentration of 3 × 10^5^ cells/ml. The cells were pelleted, and until further use, kept frozen at – 80° C. The frozen pellets were thawed on ice and suspended in lysis buffer [10 mM Tris–HCl (pH 7.5), 1 mM MgCl_2_, 300 mM NaCl and 0.2% NP40 plus yeast protease inhibitors (Sigma)]. After adding 500 units of Benzonase (Sigma E8263) extracts were rotated on a Nutator for 30 min at 4 °C. WCEs were clarified by centrifugation at 16,000 × g for 30 min. The resulting soluble material was incubated with 50μL of packed M2 agarose (Sigma) at 4 °C for at least 2 h. The M2 agarose was washed once with 10 mL IPP300 (10 mM Tris–HCl pH 8.0, 300 mM NaCl, 0.1% NP40), twice with 5 mL of IP100 buffer (10 mM Tris–HCl pH 8.0, 100 mM NaCl, 0.1% NP40), and twice with 5 mL of IP100 buffer without detergent (10 mM Tris–HCl pH 8.0, 100 mM NaCl). 500 μL of 0.5 M NH_4_OH was added to elute the proteins by rotating for 20 min at room temperature.

For tandem affinity purification performed for all analyzed baits except for the H3-GFP and CSKB1-FZZ which were subjected to one-step affinity purification described above, clarified WCEs were incubated with 250 μl packed beads volume of IgG-Sepharose chromatography resin for the first step of affinity purification for 4 h at 4 °C. Beads were washed once with IPP300 and twice with 1 × Tev buffer (10 mM Tris–HCl pH 8.0, 100 mM NaCl, 0.1% NP40, 1 mM EDTA) before being treated overnight with TEV protease. Next day, the supernatant was incubated with 50 μL of packed M2 agarose (Sigma) and the procedure was carried out as described above. Preparation of protein eluates for mass spectrometry acquisition is detailed in Additional file 3: Methods S1.

### MS data visualization and archiving

Cytoscape (V3.4.0; [81]) was used to generate protein–protein interaction networks with individual nodes manually arranged. Heatmaps and Dot plots were generated using ProHits-viz [82]. The annotation of the co-purifying partners was carried out using BLAST (https://blast.ncbi.nlm.nih.gov/Blast.cgi), as well as by performing SMART domain analysis (http://smart.embl-heidelberg.de/). All MS files used in this study were deposited at MassIVE (http://massive.ucsd.edu) and assigned the identifier MSV000090060. 

### ChIP-Seq

The ChIP experiments were performed as described previously with modifications detailed in the Additional file 3: Methods S1 [83, 84].

### Gene expression data

We used microarray data (accession number GSE11300) (http://tfgd.ihb.ac.cn/) [56] to examine the gene expression profiles.

### RNA extraction and sequencing

Total RNA was extracted from wildtype and H3.3 KO *Tetrahymena* during vegetative growth using the RNeasy extraction kit (Qiagen) following the manufacturer’s instructions. Two independent biological samples for each condition were generated. RNA was treated with DNase, and total RNA was quantified using Qubit RNA BR (cat # Q10211, Thermo Fisher Scientific Inc., Waltham, USA) fluorescent chemistry. 1000 ng per sample was processed using the NEBNext Ultra II Directional RNA Library Prep Kit for Illumina (cat # E7760L; New England Biolabs, Ipswich, USA; protocol v. v3.1_5/20), including PolyA selection. 1 uL top stock of each purified final library was analyzed on an Agilent Bioanalyzer dsDNA High Sensitivity chip (cat # 5067-4626, Agilent Technologies Inc., Santa Clara, USA). The libraries were quantified using the Quant-iT dsDNA high sensitivity (cat # Q33120, Thermo Fisher Scientific Inc., Waltham, USA) and were pooled at equimolar ratios after size adjustment. The quantified pool was hybridized at a final concentration of 2.215 pM, and single-end reads were obtained on an Illumina NextSeq 500 platform using a full High-Output v2.5 flowcell at 75 bp read lengths.

### Quantitative PCR

For RT-qPCR analysis, total RNA was extracted from the H3.3 KO or WT *Tetrahymena* cells using TRIzol (Life Technologies) according to the supplier's instructions. The isolated RNA was treated with DNase I (RNase-free, Thermo Fisher Scientific Inc., Waltham, USA). cDNA was prepared using iScript™ Reverse Transcription Supermix for RT-qPCR. qPCR was performed in technical triplicates from three individual KO cell lines. The data were normalized to the expression levels of beta Actin.

### Indirect immunofluorescence

Cells were fixed during vegetative growth, 24-h starvation, and conjugation (2, 4, 6 and 7.5 h post mixing) to perform indirect immunofluorescence as previously described [46]. Cells were washed in 10 mM Tris–HCl, pH 7.7, fixed in 4% paraformaldehyde and membrane permeabilized with cold acetone for 20 min. Incubation with primary mouse anti-FLAG antibody (or anti-IgG or anti-GFP) (Sigma) was at a 1:500 dilution at 4 °C overnight in 1 × PBST (phosphate-buffered saline with Tween 20). Cells were washed three times in 1 × PBS. 1-h incubation in secondary antibody fluorescein isothiocyanate-conjugated (FITC) goat anti-mouse (Pierce) was performed at room temperature. 4,6-diamidino-2-phenylindole dihydrochloride (DAPI) was used to stain the nuclei. Immunofluorescence analysis was carried out using an Olympus DP70 equipped with a fluorescent microscope (Reichert-Jung, POLYVER) at 100× magnification without oil. Image preparation was performed using ImageJ (1.50i) software [85].

### AlphaFold structural predictions and molecular modeling

The complete open-source version of DeepMind’s AlphaFold V2.1.0 tool was used to fold and model each full-length protein [57, 86], and a recent extension to AlphaFold 2, AlphaFold-Multimer, was used to model heterodimers of each protein–protein pair to predict structural contexts of their putative interactions [87]. TTHERM_00442300, TTHERM_00219420, and TTHERM_00046490 protein sequences were selected for AlphaFold input, representing *Tetrahymena* Asf1, Cac2, and Hir1 homologs, respectively.

When predicting the co-structure of each protein pair, residues experimentally determined as important for interaction were not provided to AlphaFold, allowing for an unbiased prediction of interaction surfaces. AlphaFold model parameters under Creative Commons Attribution 4.0 license were used for modeling of both individual proteins and co-structures [57]. In addition to AlphaFold, mmseq2 [88] was used for Multiple Sequence Alignment (MSA) prior to prediction, and Amber was used to refine side-chain bond geometry following prediction [89]. Highest-confidence models of individual proteins and heterogeneous co-structures were selected by their pLDDT and pTMscores, respectively, as calculated with AlphaFold. All visualizations were generated with Pymol.

## Quantification and statistical analysis

### ChIP-seq analysis

ChIP-seq analysis was performed essentially as previously described [48]. Briefly, Illumina adaptor sequences were removed from the 3′ ends of 51-nt reads, and the remaining reads were mapped to the *Tetrahymena* genome using STAR (ver 2.7.1) with default settings.

After removal of duplicate reads, peaks were called jointly on immunoprecipitated and input samples with MACS2 (version 2.1.2) [90]. The metagene analysis was performed using ChIP-Seq reads normalized over the inputs and by ‘Reads Per Kilobase of transcript per Million mapped reads (RPKM)’ values. The plots were generated using deeptools [91].

### RNA-seq analysis

To identify the differentially expressed genes from RNA-seq data, we used DESeq2 (ver 3.11) [92] on gene counts generated using STAR and the *Tetrahymena* genome annotation (T_Thermophila_MAC_2021-Updated Gene Names). We filtered out genes with less than 10 counts across the sum of all RNA-seq samples. To plot differentially expressed genes as volcano plots, R-package EnhancedVolcanoplot (https://github.com/kevinblighe/EnhancedVolcano) was used. GO/KEGG enrichment was performed using ShinyGO (v0.61), which utilizes a hypergeometric distribution followed by FDR correction, where the FDR cut-off was set to 0.05.

### Data deposition

All MS files generated in this study were deposited at MassIVE (http://massive.ucsd.edu) and assigned the identifier MSV000090060. ChIP-seq and RNA-seq data generated can be found online at Gene Expression Omnibus (GEO, https://www.ncbi.nlm.nih.gov/geo/) with unique identifier GSE210903. 

## Supplementary Information

**Additional file 1: Table S1.** AP-MS analysis of H3. **Table S2.** AP-MS analysis of H3.3. **Table S3.** AP-MS analysis of Nrp1. **Table S4.** AP-MS analysis of Aip1. **Table S5.** AP-MS analysis of HIR1. **Table S6.** AP-MS analysis of Cac2. **Table S7.** AP-MS analysis of CSKB1. **Table S8.** AP-MS analysis of HAT1. **Table S9.** H3.3 ChIP peaks file. **Table S10.** H3.3 Knockout RNA-seq. **Table S11.** Primer sequences. **Table S12.** READ ME: Details of mass spectrometry files deposition to the MassIVE repository.

**Additional file 2: Figure S1.** Endogenous tagging of H3 and H3.3 in *Tetrahymena*. **A: **Comparison of *Tetrahymena* H3 variants and histone chaperones’ nomenclature with human gene/protein names.** B: **Multiple sequence alignment showing the conservation of *Tetrahymena* H3 and H3.3. 15 residues vary between H3 and H3.3. Conservation score key is provided. **B: **Schematic depiction of epitope tagging strategy for the MAC locus. **C: **Indirect immunofluorescence analysis of H3GFP and H3.3-FZZ in growing *Tetrahymena*. DAPI was used to stain the nuclei and the position of the MAC and MIC is indicated with arrows and arrowheads, respectively. Untagged wildtype *Tetrahymena* were used as a control. **Figure S2.** Aip1 shows cytoplasmic localization in growing *Tetrahymena*. **A: **Western blotting analysis using whole cell lysates prepared from vegetative *Tetrahymena* cells expressing Aip1FZZ. The blots were probed with the indicated antibodies.** B:** Indirect immunofluorescence analysis of Nrp1-GFP in growing *Tetrahymena*. Untagged wildtype *Tetrahymena* were used as a control. **C:** Indirect immunofluorescence analysis of Aip1-FZZ in starved *Tetrahymena* cells.** D: **Indirect immunofluorescence analysis of macronuclear Hv1-FZZ (left) and micronuclear linker histone Mlh1-FZZ (right) in growing *Tetrahymena*. Note: DAPI was used to stain the nuclei and the positions of the MAC and MIC are indicated with arrows and arrowheads, respectively. **Figure S3.** H3 (H3.3)/H4 chaperones show similar expression profiles. Heatmap representation of microarray expression values for Asf1, Hir1, Cac2, and Nrp1. Z scores were calculated across the rows for each gene to examine its differential expression across growth, starvation, and developmental stages. L1–LH: Logarithmic growth phase, S0–24: Starvation for 24 h, C: Conjugation where 0–18 are hours post mixing the different mating types. Hierarchical clustering was used to examine the expression profiles. **Figure S4.** Asf1^Tt^ structure is conserved in *Tetrahymena*. **A)** AlphaFold-predicted structure of TTHERM_00442300 (Asf1^Tt^) **B) **Model alignment comparison of Asf1^Tt^ from this study with the Human ASF1A homolog model generated by the AlphaFold Deepmind consortium (AlphaFold Database ID: Q9Y294). Asf1^Tt^ is colored in green; Human ASF1A is colored in magenta. **C) **Predicted structure of Asf1^Tt^ colored by pLDDT per residue confidence score ranging from orange (very low: pLDDT<50) to dark blue (very high: pLDDT>90). **D) **Model alignment comparison of Asf1^Tt^ from this study with Human ASF1A colored by pLDDT score. **Figure S5.** Cac2^Tt^ forms β-propeller-like structure. AlphaFold-predicted structure of TTHERM_00442300 (Cac2^Tt^) protein depicting side **(A)** and top **(B)** views respective to the βpropeller motif. The B-domain of Cac2^Tt^ is colored in red. **Figure S6.** Hir1^Tt^ structure prediction. AlphaFold-predicted structure of TTHERM_00046490 (Hir1^Tt^) protein depicting side **(A) **and top **(B)** views respective to the β-Propeller motif. The B-domain of Hir1^Tt^ is colored in red. **Figure S7.** Protein complex prediction of Cac2^Tt^ and Asf1^Tt^. Overall AlphaFold-predicted structure of TTHERM_00219420 (Cac2^Tt^) bound with TTHERM_00442300 (Asf1^Tt^). Cac2^Tt^ is colored in cyan and Asf1^Tt^ is colored green. The B-domain of Cac2^Tt^ is colored in red. **Figure S8.** Visualization of the predicted binding interface between Cac2^Tt^ and Asf1^Tt^. Cac2^Tt^ is colored in cyan, whereas Asf1^Tt^ is colored green and the B-domain of Cac2^Tt^ is highlighted in red. Labeled residues (K87-G531, D89-K534, R146-D372) are predicted to form polar intermolecular contacts between Asf1^Tt^ and Cac2^Tt^ within 3Å, and an intramolecular π interaction (F393-K535) involving a lysine residue within the B-domain of Cac2^Tt^ (T527-Y545). All interactions are shown as dashed yellow lines and arrows. **Figure S9. **Visualization of the predicted binding interface between Hir1^Tt^ and Asf1^Tt^. **A:** AlphaFold-predicted co-structure of TTHERM_00046490 (Hir1^Tt^) with Asf1^Tt^. Hir1^Tt^ is colored in gray, Asf1^Tt^ is colored green. The B-domain of Hir1^Tt^ is colored in red. No significant intermolecular interactions were detected in our predictions. **B: Left, **AlphaFold-predicted co-structure. Hir1^Tt^ is colored by pLDDT per residue confidence scores ranging from orange (very low: pLDDT<50) to dark blue (very high: pLDDT>90). Asf1^Tt^ is colored in green. **Right**: AlphaFold-predicted by-residue pLDDT confidence score plot for the 5 highest-confidence Hir1^Tt^ models. All models display low predictive confidence for residues of the Hir1^Tt^ B-Domain (res. 453:476) and directly up and downstream of the B-domain. **Figure S10.** Endogenous tagging of *Tetrahymena* Hat1. **A: **Western blotting analysis using whole cell lysates prepared from vegetative *Tetrahymena* cells expressing Hat1-FZZ. The blots were probed with the indicated antibodies. **B:** Indirect immunofluorescence analysis of Hat1-FZZ in growing *Tetrahymena*. DAPI was used to stain the nuclei, and the positions of the MAC and MIC are indicated with arrows and arrowheads, respectively. Untagged wildtype *Tetrahymena* were used as a control. **Figure S11.** Strategy to confirm the correct integration of *NEO* cassette. **Top**, Schematic representation of the confirmation of the accurate integration of the *NEO* cassette at the locus of interest. Positions of PCR primers are indicated. The reverse primer is designed complementary to sequence within the *NEO *cassette, whereas the forward primer is specific to sequence upstream of the gene of interest. A PCR product will be observed only if the NEO cassette is integrated into the desired locus. WT cells will not show PCR products. Primers designed to amplify DNA from the promoter regions of each target gene were used as loading controls. **Bottom**, Agarose gel electrophoresis using genomic DNA extracted either from KO or WT *Tetrahymena* cells. **Figure S12.** KO analysis of *CAC2*^*Tt*^ and *HIR1*^*Tt*^ in *Tetrahymena*. A: Left, Fluorescence (DAPI) of vegetative and starved *∆CAC2 *and wildtype *Tetrahymena* cells. **Right**, Bar plots showing the quantification of mean diameter of MACs in *∆CAC2*^*Tt*^ compared to wildtype *Tetrahymena*. Diameters were measured in micrometers for 40 individual *Tetrahymena* cells. Images used were taken at 40X magnification in a 1360x1024 frame. Field of view at 40X was 360 micrometers. **B: **Fluorescence (DAPI) analysis of conjugating wildtype, Δ*HIR1*^*Tt*^, and Δ*Cac2*^*Tt*^ *Tetrahymena* cells. Hours post mixing the *Tetrahymena* cells of different mating types are indicated on the left. Note: DAPI was used to stain the nuclei.** Figure S13.** Indirect immunofluorescence analysis in starved *Tetrahymena*. Indirect immunofluorescence analysis in starved *Tetrahymena*. **A: **Expression profile of Hir1 during growth and starvation in *Tetrahymena*. For growing cells, L-l corresponds to ~1X10^5^ cells/mL. For starvation, ~2X105 cells/mL were collected at 0, 3, 6, 9, 12, 15, and 24 hours referred to as S-0, S-3, S-6, S-9, S-12, S-15, and S-24. Microarray data was acquired from http://tfgd.ihb.ac.cn/search/detail/gene/TTHERM_00046490 (last accessed January 20, 2023) **B: **Indirect immunofluorescence analysis of H3.3-FZZ in starved *Tetrahymena*. H3.3 is found in the MAC only. **C: **Indirect immunofluorescence analysis of RebL1-FZZ in starved *Tetrahymena*. RebL1 is found in the MAC only. DAPI was used to stain the nuclei. The positions of the MAC and MIC are indicated with arrows and arrowheads, respectively. **Figure S14.** H3.3 ChIP-seq replicates correlate with each other. **A: **Principal component analysis (PCA) of two H3.3 ChIP-seq replicates and their corresponding inputs. **B: **Fingerprint plot to examine the quality of H3.3 ChIP signal in comparison with inputs. ChIP-seq is enriched as more reads are found in smaller number of bins for ChIPs compared to the input. **Figure S15.** GO enrichment analysis of H3.3 ChIP-seq targets. **A: **Bar plot depicts the % overlap of upregulated and downregulated genes in H3.3 KO cells with those genes classified as highly expressed during *Tetrahymena* vegetative growth. **B: **KEGG pathway enrichment analysis of H3.3 bound genes. Number of genes for each term is indicated beside each bar. **C: **Dot plot representation of pfam domain enrichment analysis in H3.3-target genes (Q< 0.05). Figure legend is provided. **Figure S16.** Predicted CKII sites on Cac2^Tt^ and Cac1^Tt^ proteins. The red box shows the conserved sequence, whereas star indicates the serine residue predicted to be phosphorylated by CKII. The prediction was performed using Netphos web server https://services.healthtech.dtu.dk/services/NetPhos-3.1/.

**Additional file 3: **Methods S1.

## Data Availability

The data that support the findings of this study are available from the corresponding author upon reasonable request.
