# Supplementary material for: Multilevel interrogation of H3.3 reveals a primordial role in transcription regulation
Source: Epigenetics Chromatin. 2023 Apr 7;16:10. doi: 10.1186/s13072-023-00484-9 (PMC10080907; doi:10.1186/s13072-023-00484-9)
Supplement: Supplementary file 2 — Additional file 2: Figure S1. Endogenous tagging of H3 and H3.3 in Tetrahymena. A: Comparison of Tetrahymena H3 variants and histone chaperones’ nomenclature with human gene/protein names. B: Multiple sequence alignment showing the conservation of Tetrahymena H3 and H3.3. 15 residues vary between H3 and H3.3. Conservation score key is provided. B: Schematic depiction of epitope tagging strategy for the MAC locus. C: Indirect immunofluorescence analysis of H3GFP and H3.3-FZZ in growing Tetrahymena. DAPI was used to stain the nuclei and the position of the MAC and MIC is indicated with arrows and arrowheads, respectively. Untagged wildtype Tetrahymena were used as a control. Figure S2. Aip1 shows cytoplasmic localization in growing Tetrahymena. A: Western blotting analysis using whole cell lysates prepared from vegetative Tetrahymena cells expressing Aip1FZZ. The blots were probed with the indicated antibodies. B: Indirect immunofluorescence analysis of Nrp1-GFP in growing Tetrahymena. Untagged wildtype Tetrahymena were used as a control. C: Indirect immunofluorescence analysis of Aip1-FZZ in starved Tetrahymena cells. D: Indirect immunofluorescence analysis of macronuclear Hv1-FZZ (left) and micronuclear linker histone Mlh1-FZZ (right) in growing Tetrahymena. Note: DAPI was used to stain the nuclei and the positions of the MAC and MIC are indicated with arrows and arrowheads, respectively. Figure S3. H3 (H3.3)/H4 chaperones show similar expression profiles. Heatmap representation of microarray expression values for Asf1, Hir1, Cac2, and Nrp1. Z scores were calculated across the rows for each gene to examine its differential expression across growth, starvation, and developmental stages. L1–LH: Logarithmic growth phase, S0–24: Starvation for 24 h, C: Conjugation where 0–18 are hours post mixing the different mating types. Hierarchical clustering was used to examine the expression profiles. Figure S4. Asf1Tt structure is conserved in Tetrahymena. A) AlphaFold-predicted [file 13072_2023_484_MOESM2_ESM.pdf]

Figure S1

A

Tetrahymena name	Common name	Remarks
HHT1 and HHT2	Histone H3	Replication-dependent (RD) histone H3
HHT3	H3.3	Replication independent (RI) H3 variant
HHT4	Similar to H3.3	Replication independent H3 variant; should not be confused with testis-specific H3.4 found in animals.
Nrp1	NASP	Generalized H3(H3.3)/H4 chaperone
RebL1	RBBP4/RBBP7	H3(H3.3)/H4 chaperone; component of various chromatin modifying complexes, e.g., HAT1, CAF1, SIN3
Hir1	HirA	RI H3.3-specific chaperone
Cac2	Chaf1b (Cac2)	RD H3-specific chaperone; component of CAF1-complex
Asf1	Asf1	Generalized H3(H3.3)/H4 chaperone

B

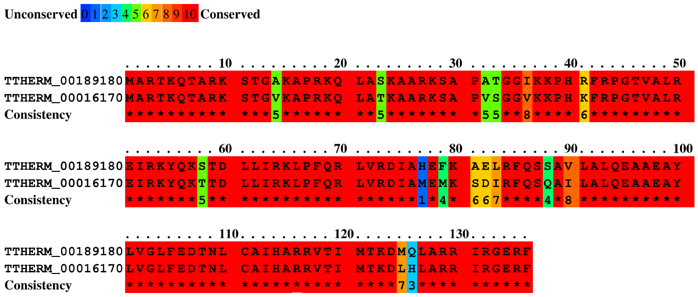

C

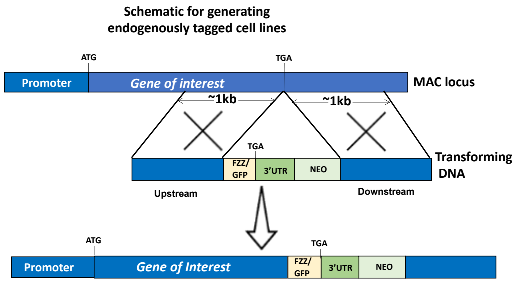

D

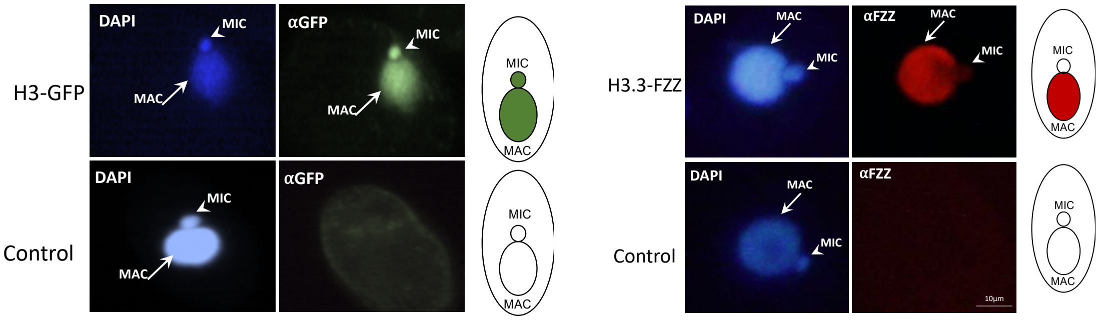

Figure S1: Endogenous tagging of H3 and H3.3 in *Tetrahymena*. A: Comparison of *Tetrahymena* H3 variants and histone chaperones' nomenclature with human gene/protein names. **B:** Multiple sequence alignment showing the conservation of *Tetrahymena* H3 and H3.3. 15 residues vary between H3 and H3.3. Conservation score key is provided. **B:** Schematic depiction of epitope tagging strategy for the MAC locus. **C:** Indirect immunofluorescence analysis of H3-GFP and H3.3-FZZ in growing *Tetrahymena*. DAPI was used to stain the nuclei and the position of the MAC and MIC is indicated with arrows and arrowheads, respectively. Untagged wildtype *Tetrahymena* were used as a control.

Figure S2

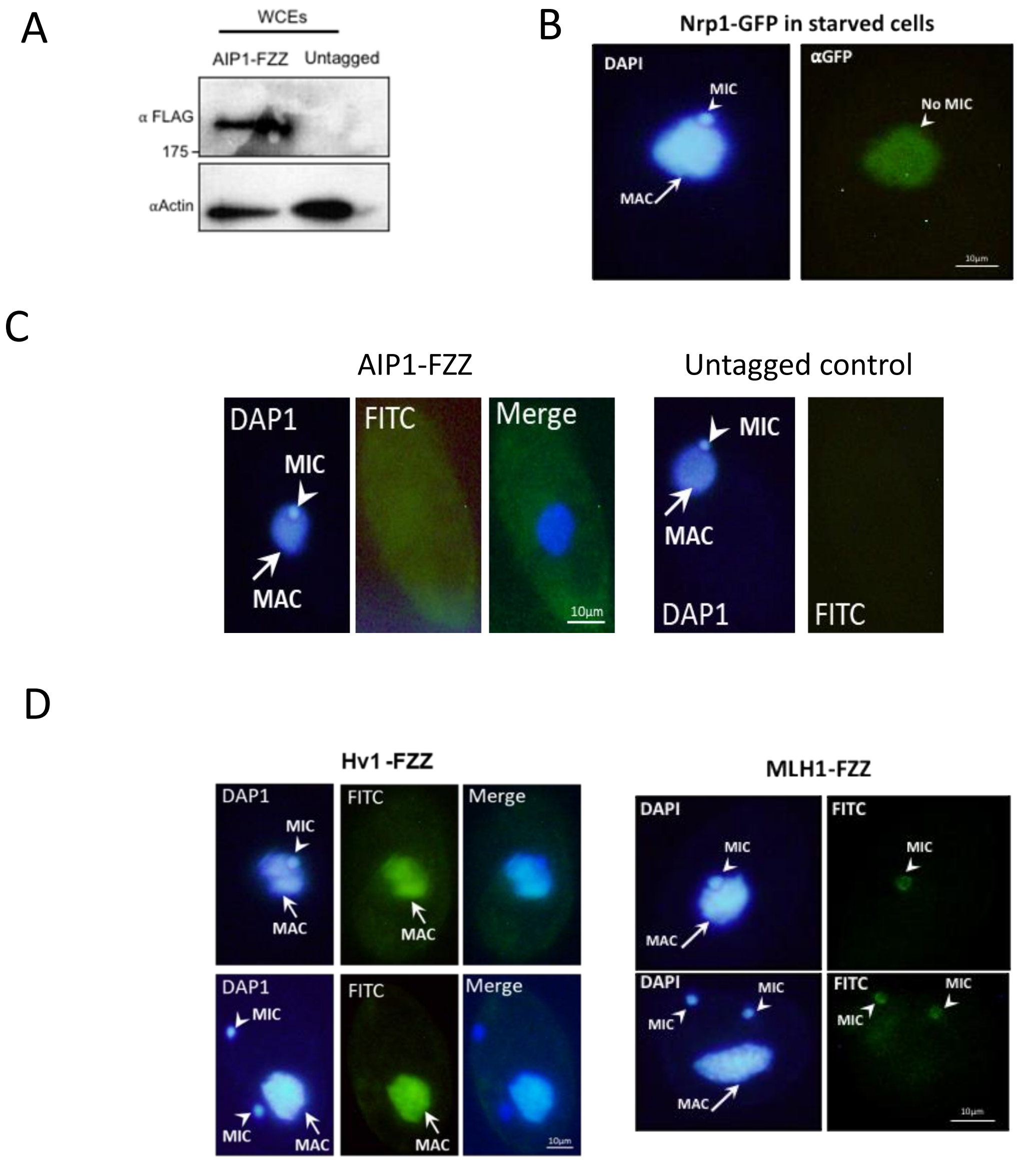

Figure S2: Aip1 shows cytoplasmic localization in growing *Tetrahymena*. A: Western blotting analysis using whole cell lysates prepared from vegetative *Tetrahymena* cells expressing Aip1-FZZ. The blots were probed with the indicated antibodies. **B:** Indirect immunofluorescence analysis of Nrp1-GFP in growing *Tetrahymena*. Untagged wildtype *Tetrahymena* were used as a control. **C:** Indirect immunofluorescence analysis of Aip1-FZZ in starved *Tetrahymena* cells. **D:** Indirect immunofluorescence analysis of macronuclear Hv1-FZZ (left) and micronuclear linker histone Mlh1-FZZ (right) in growing *Tetrahymena*. Note: DAPI was used to stain the nuclei and the positions of the MAC and MIC are indicated with arrows and arrowheads, respectively.

Figure S3

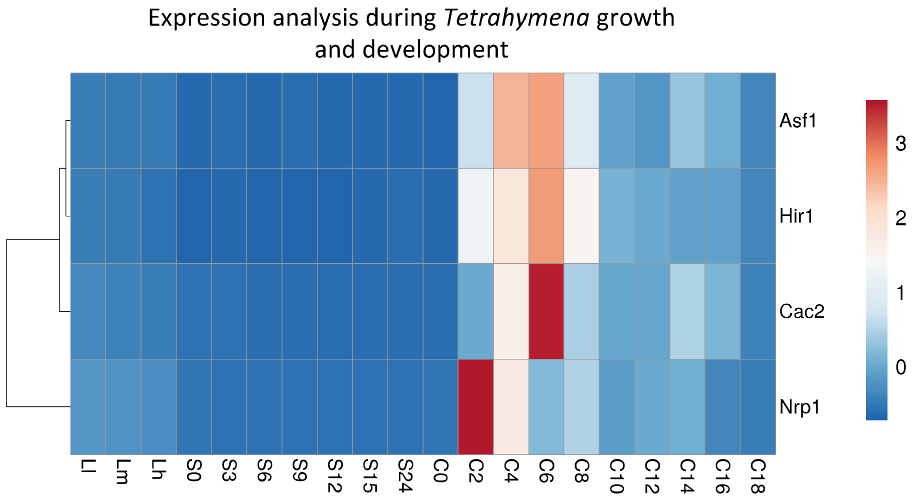

Figure S3: H3 (H3.3)/H4 chaperones show similar expression profiles. Heatmap representation of microarray expression values for Asf1, Hir1, Cac2 and Nrp1. Z scores were calculated across the rows for each gene to examine its differential expression across growth, starvation, and developmental stages. L1–LH: Logarithmic growth phase, S0–24: Starvation for 24 h, C: Conjugation where 0–18 are hours post mixing the different mating types. Hierarchical clustering was used to examine the expression profiles.

Figure S4

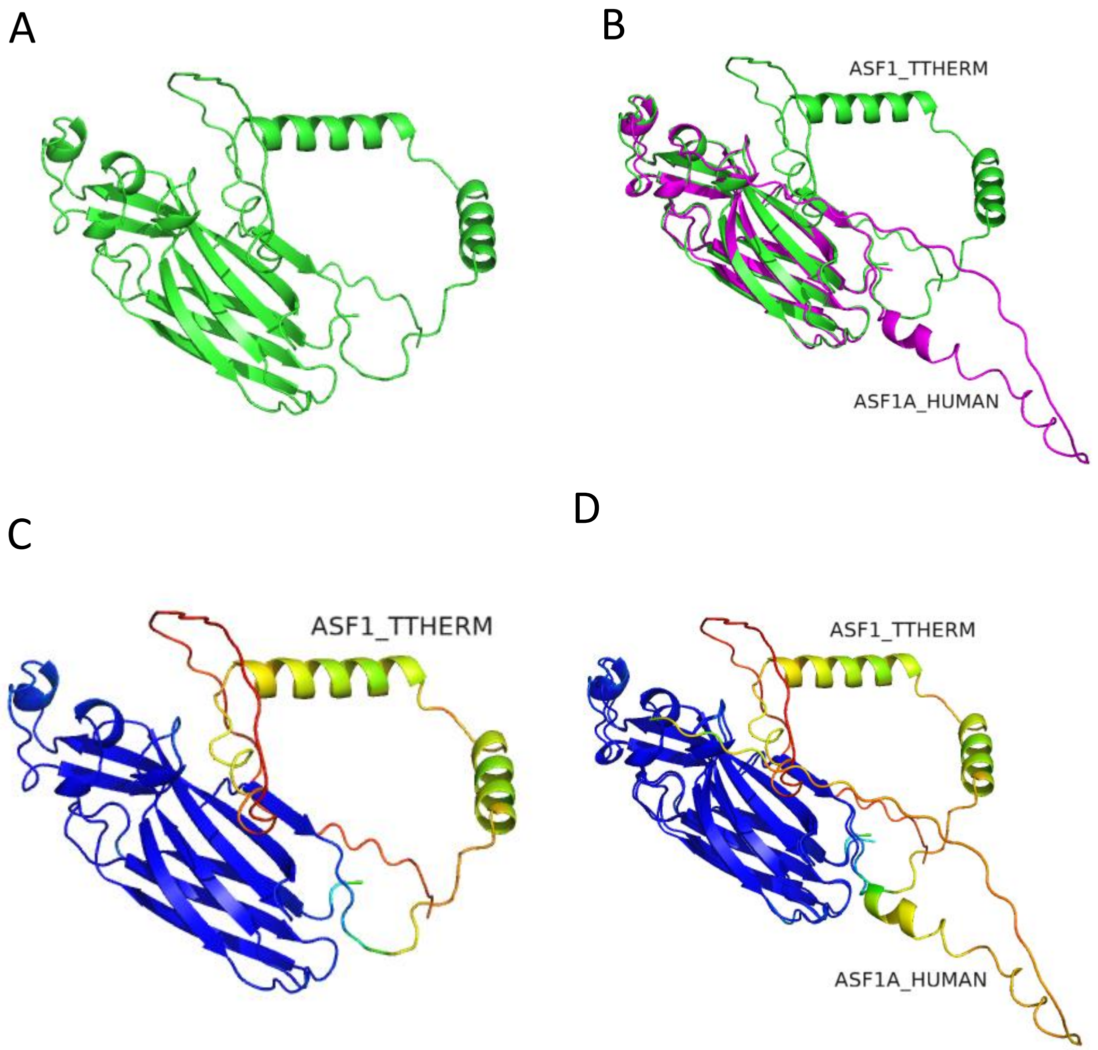

Figure S4: Asf1^{Tt} structure is conserved in *Tetrahymena*. A) AlphaFold-predicted structure of TTHERM_00442300 (Asf1^{Tt}) **B)** Model alignment comparison of Asf1^{Tt} from this study with the Human ASF1A homolog model generated by the AlphaFold Deepmind consortium (AlphaFold Database ID: Q9Y294). Asf1^{Tt} is colored in green, Human ASF1A is colored in magenta. **C)** Predicted structure of Asf1^{Tt} colored by pLDDT per residue confidence score ranging from orange (very low: pLDDT<50) to dark blue (very high: pLDDT>90). **D)** Model alignment comparison of Asf1^{Tt} from this study with Human ASF1A coloured by pLDDT score.

Figure S5

A) SIDE VIEW

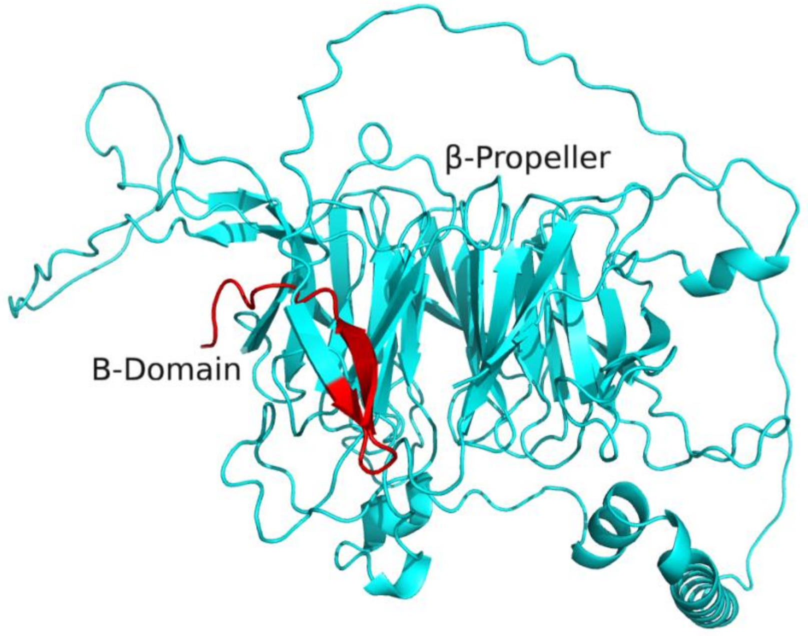

B) TOP VIEW

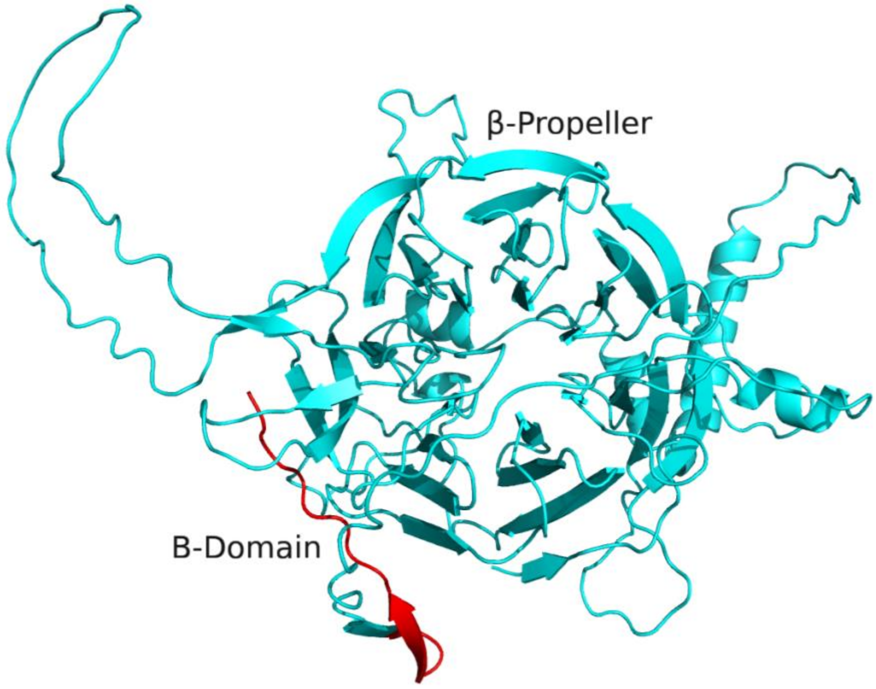

Figure S5: Cac2^{Tt} forms β-propeller like structure. AlphaFold-predicted structure of TTHERM_00442300 (Cac2^{Tt}) protein depicting side **(A)** and top **(B)** views respective to the β-propeller motif. The B-domain of Cac2^{Tt} is colored in red.

Figure S6

A) SIDE VIEW

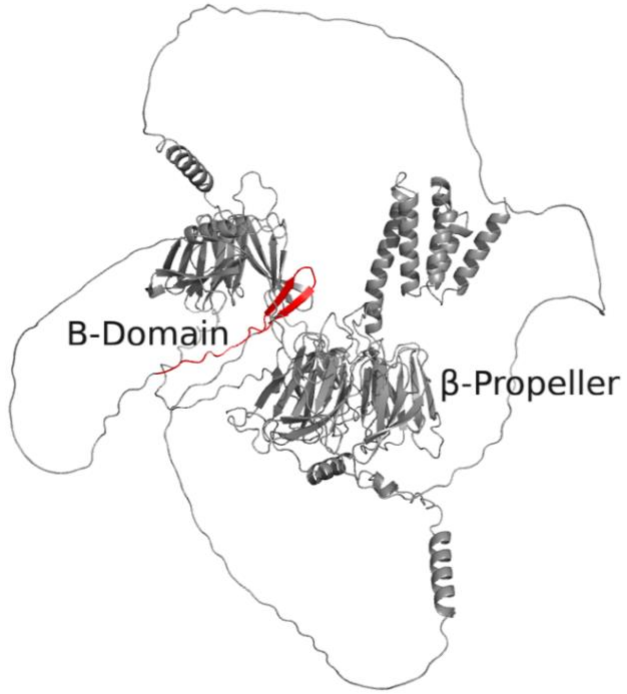

B) TOP VIEW

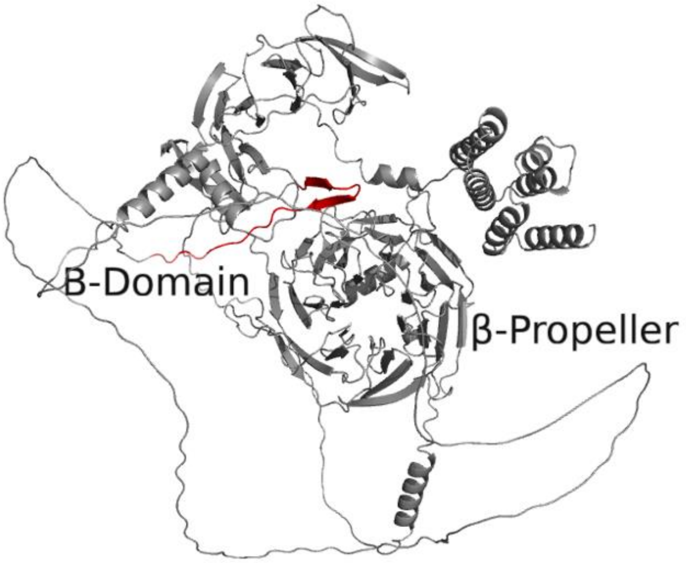

Figure S6: Hir1^{Tt} structure prediction. AlphaFold-predicted structure of TTHERM_00046490 (Hir1^{Tt}) protein depicting side **(A)** and top **(B)** views respective to the β-Propeller motif. The B-domain of Hir1^{Tt} is colored in red.

Figure S7

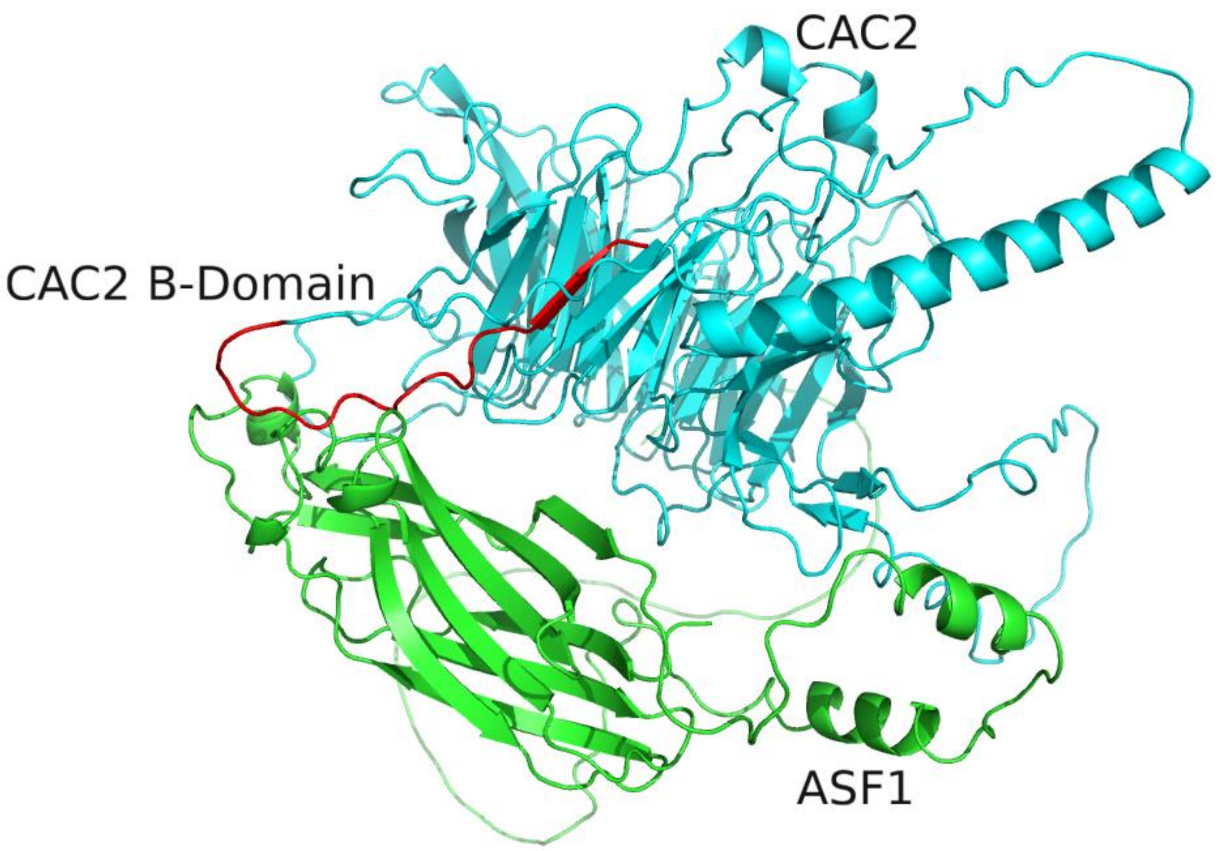

Figure S7: Protein complex prediction of Cac2^{Tt} and Asf1^{Tt}. Overall AlphaFold-predicted structure of TTHERM_00219420 (Cac2^{Tt}) bound with TTHERM_00442300 (Asf1^{Tt}). Cac2^{Tt} is colored in cyan and Asf1^{Tt} is colored green. The B-domain of Cac2^{Tt} is colored in red.

Figure S8

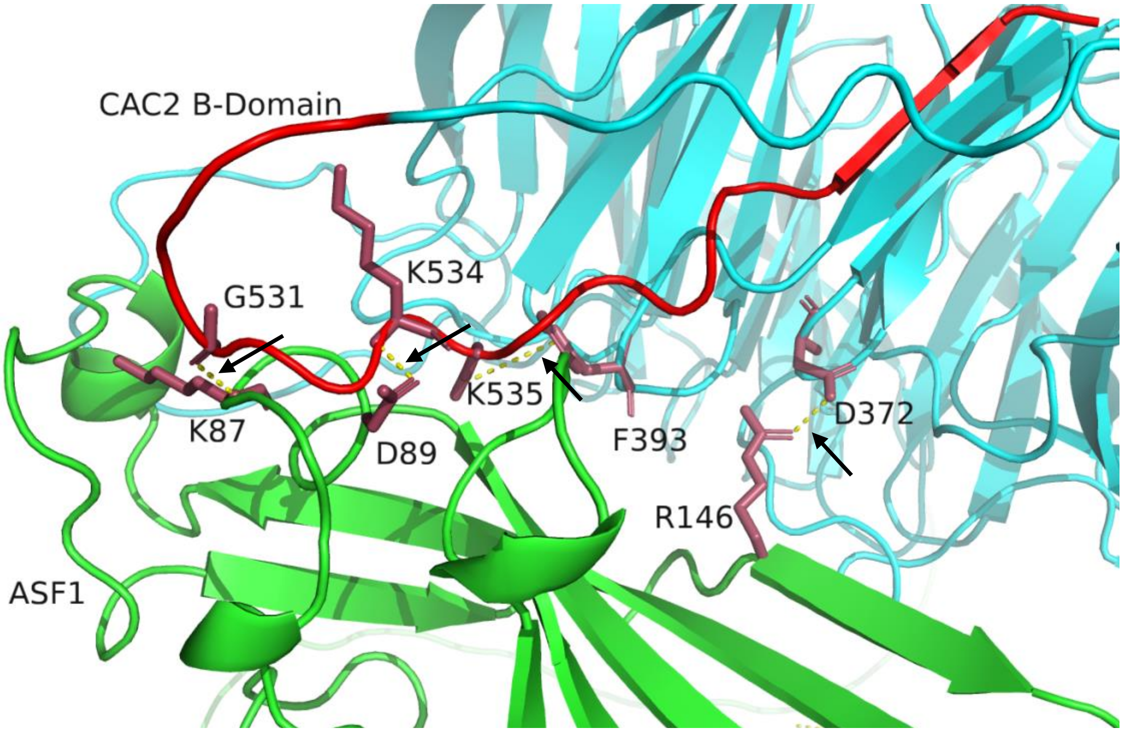

Figure S8: Visualization of the predicted binding interface between Cac2^{Tt} and Asf1^{Tt}. Cac2^{Tt} is colored in cyan whereas Asf1^{Tt} is colored green and the B-domain of Cac2^{Tt} is highlighted in red. Labelled residues (K87-G531, D89-K534, R146-D372) are predicted to form polar intermolecular contacts between Asf1^{Tt} and Cac2^{Tt} within 3Å, and an intramolecular π interaction (F393-K535) involving a lysine residue within the B-domain of Cac2^{Tt} (T527-Y545). All interactions are shown as dashed yellow lines and arrows.

Figure S9

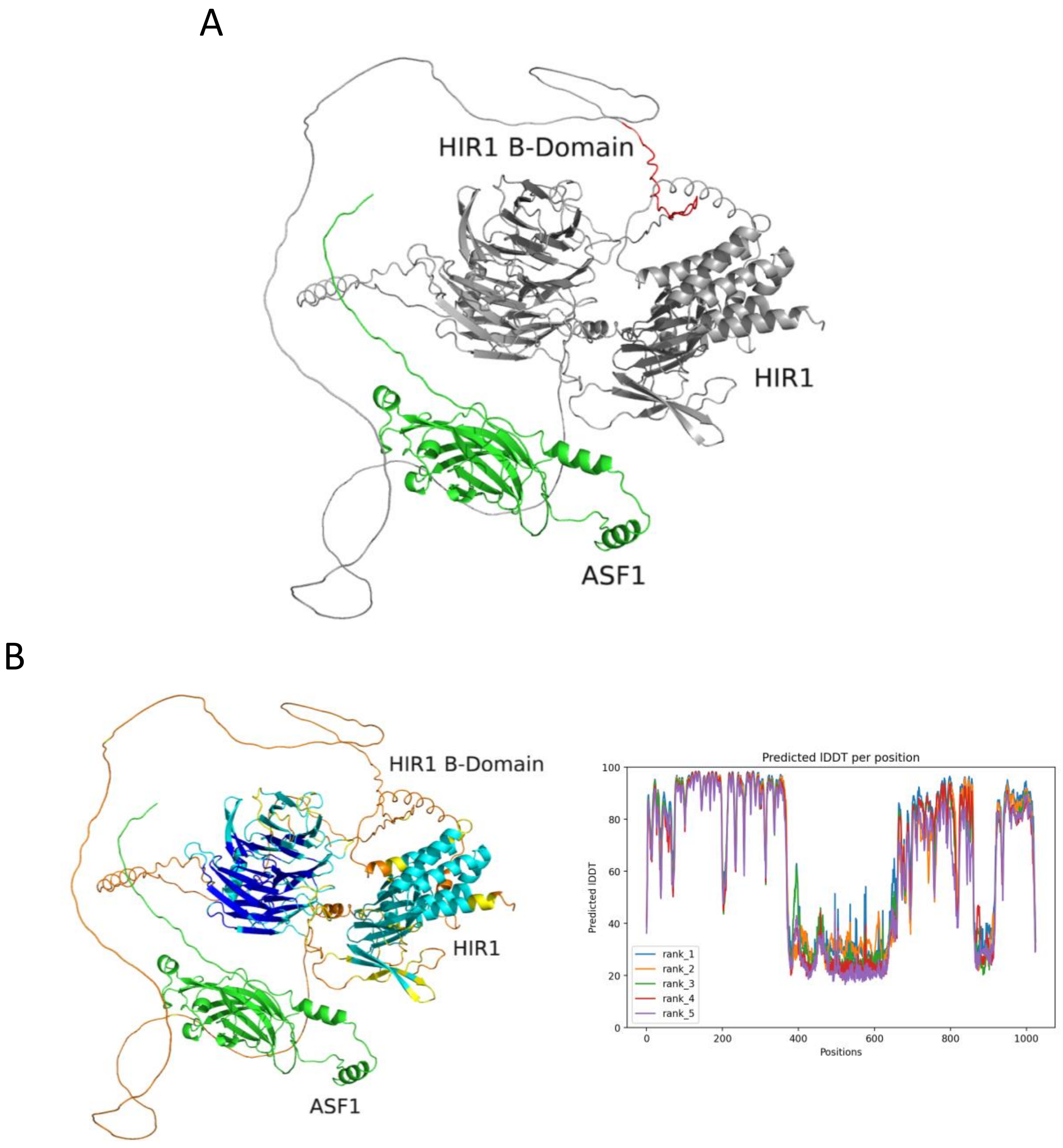

Figure S9: Visualization of the predicted binding interface between Hir1^{Tt} and Asf1^{Tt}. A: AlphaFold-predicted co-structure of TTHERM_00046490 (Hir1^{Tt}) with Asf1^{Tt}. Hir1^{Tt} is coloured in grey, Asf1^{Tt} is coloured green. The B-domain of Hir1^{Tt} is coloured in red. No significant intermolecular interactions were detected in our predictions. **B: Left,** AlphaFold-predicted co-structure. Hir1^{Tt} is coloured by pLDDT per residue confidence scores ranging from orange (very low: pLDDT<50) to dark blue (very high: pLDDT>90). Asf1^{Tt} is coloured green. **Right**: AlphaFold-predicted by-residue pLDDT confidence score plot for the 5 highest-confidence Hir1^{Tt} models. All models display low predictive confidence for residues of the Hir1^{Tt} B-Domain (res. 453:476) and directly up and downstream of the B-domain.

Figure S10

A

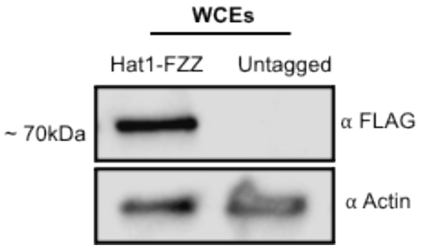

B

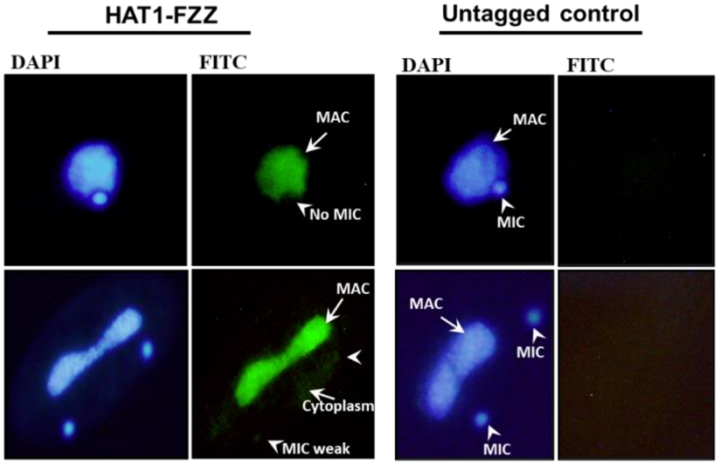

Figure S10: Endogenous tagging of *Tetrahymena* Hat1. A: Western blotting analysis using whole cell lysates prepared from vegetative *Tetrahymena* cells expressing Hat1-FZZ. The blots were probed with the indicated antibodies. **B:** Indirect immunofluorescence analysis of Hat1-FZZ in growing *Tetrahymena*. DAPI was used to stain the nuclei, and the positions of the MAC and MIC are indicated with arrows and arrowheads, respectively. Untagged wildtype *Tetrahymena* were used as a control.

Figure S11

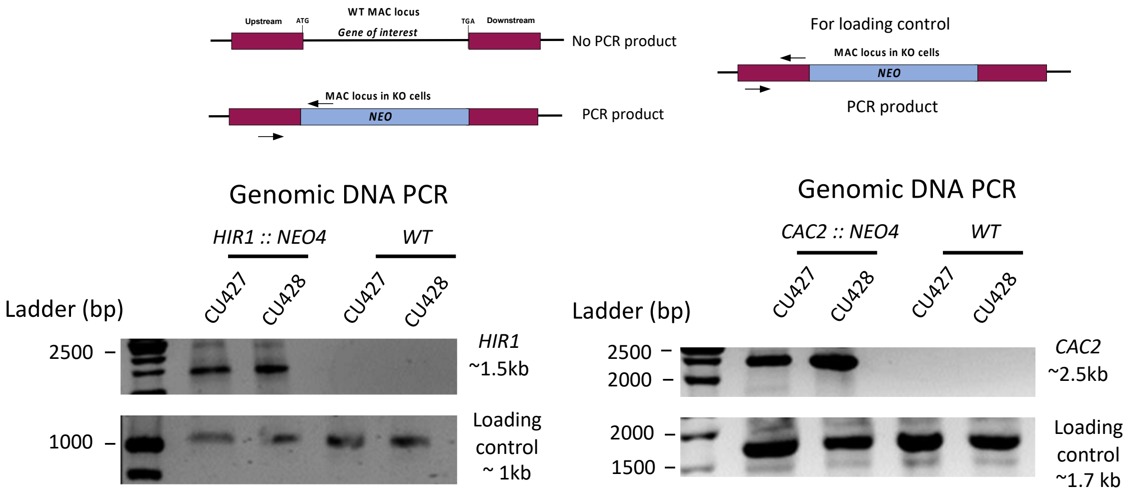

Figure S11: Strategy to confirm the correct integration of *NEO* cassette. Top, Schematic representation of the confirmation of the accurate integration of the *NEO* cassette at the locus of interest. Positions of PCR primers are indicated. The reverse primer is designed complementary to sequence within the *NEO* cassette, whereas the forward primer is specific to sequence upstream of the gene of interest. A PCR product will be observed only if the NEO cassette is integrated into the desired locus. WT cells will not show PCR products. Primers designed to amplify DNA from the promoter regions of each target gene were used as loading controls. **Bottom**, Agarose gel electrophoresis using genomic DNA extracted either from KO or WT *Tetrahymena* cells.

Figure S12

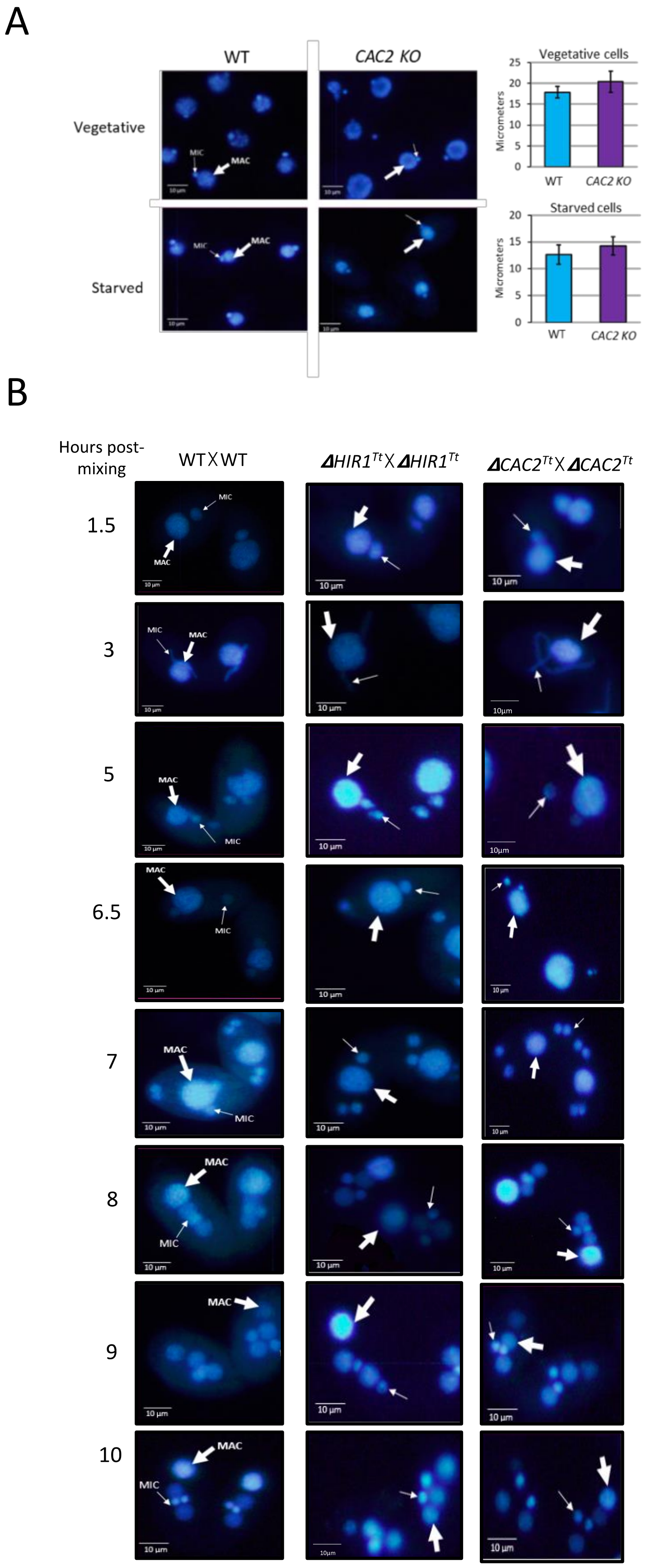
A
WT
CAC2 KO
Vegetative
Starved
MIC
MAC
Vegetative cells
Starved cells
Micrometers
WT
CAC2 KO
B
Hours post-mixing
WT X WT
ΔHIR1^Tt X ΔHIR1^Tt
ΔCAC2^Tt X ΔCAC2^Tt
1.5
3
5
6.5
7
8
9
10
MIC
MAC
10 µm

Figure S12: KO analysis of *CAC2^{Tt}* and *HIR1^{Tt}* in *Tetrahymena*. A: Left, Fluorescence (DAPI) of vegetative and starved Δ*CAC2* and wildtype *Tetrahymena* cells. **Right**, Bar plots showing the quantification of mean diameter of MACs in Δ*CAC2^{Tt}* compared to wildtype *Tetrahymena*. Diameters were measured in micrometers for 40 individual *Tetrahymena* cells. Images used were taken at 40X magnification in a 1360x1024 frame. Field of view at 40X was 360 micrometers. **B:** Fluorescence (DAPI) analysis of conjugating wildtype, ***Δ****HIR1^{Tt}*, and ***Δ****Cac2^{Tt}* *Tetrahymena* cells. Hours post mixing the *Tetrahymena* cells of different mating types are indicated on the left. Note: DAPI was used to stain the nuclei.

Figure S13

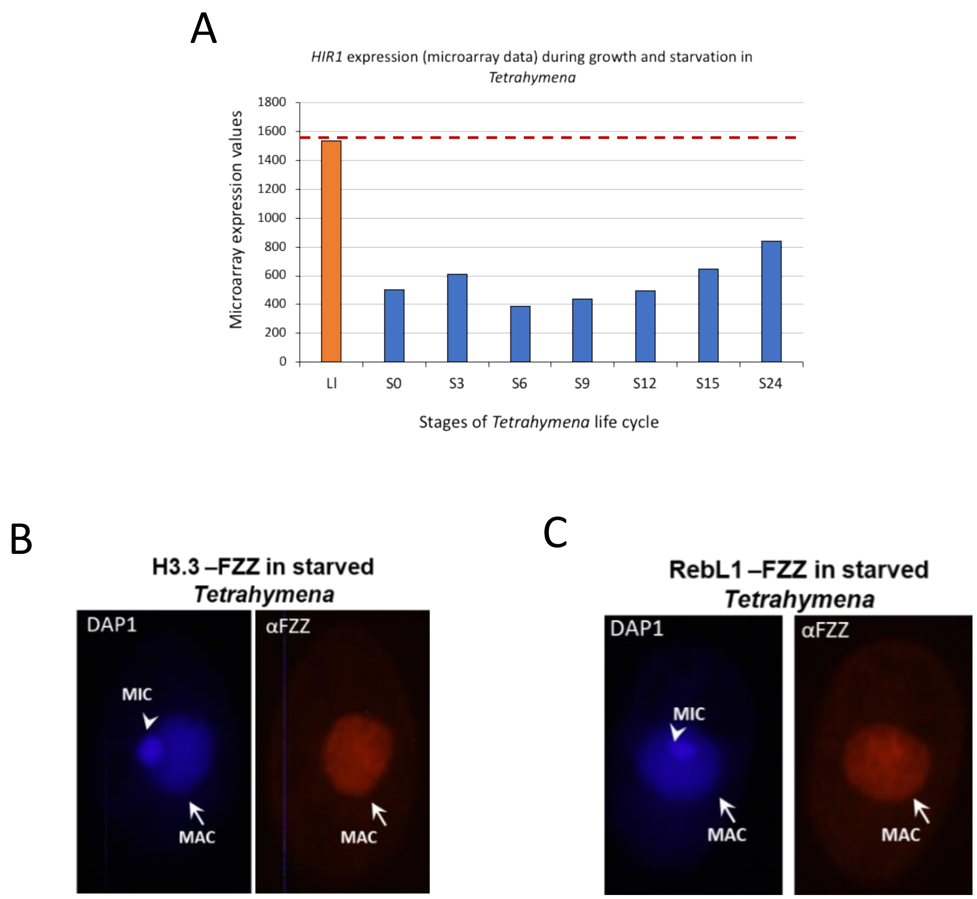

Figure S13: Indirect immunofluorescence analysis in starved *Tetrahymena*. Indirect immunofluorescence analysis in starved *Tetrahymena*. A: Expression profile of Hir1 during growth and starvation in *Tetrahymena*. For growing cells, L-l corresponds to ~1X10^5 cells/mL. For starvation, ~2X105 cells/mL were collected at 0, 3, 6, 9, 12, 15 and 24 hours referred to as S-0, S-3, S-6, S-9, S-12, S-15 and S-24. Microarray data was acquired from http://tfgd.ihb.ac.cn/search/detail/gene/TTHERM_00046490 (last accessed January 20, 2023) **B:** Indirect immunofluorescence analysis of H3.3-FZZ in starved *Tetrahymena*. H3.3 is found in the MAC only. **C:** Indirect immunofluorescence analysis of RebL1-FZZ in starved *Tetrahymena*. RebL1 is found in the MAC only. DAPI was used to stain the nuclei. The positions of the MAC and MIC are indicated with arrows and arrowheads, respectively.

Figure S14

A

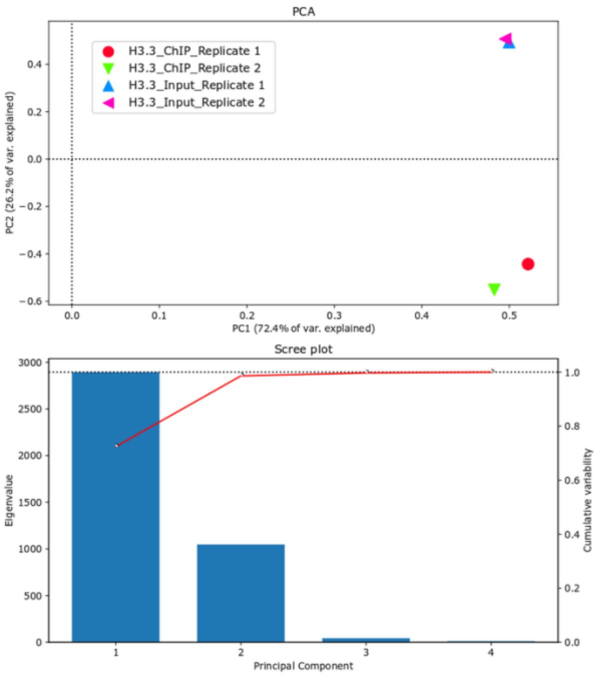

B

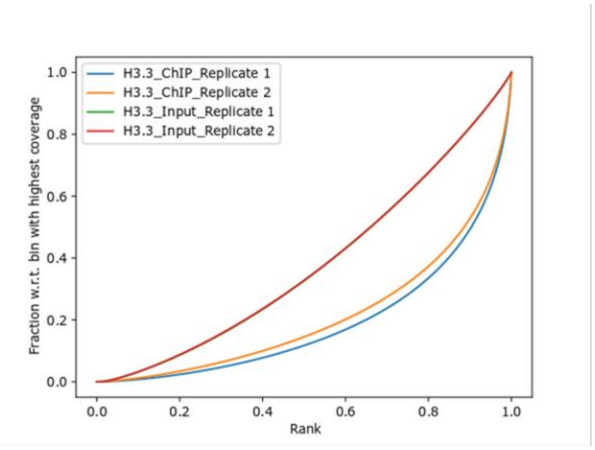

Figure S14: H3.3 ChIP-seq replicates correlate with each other. A: Principal component analysis (PCA) of two H3.3 ChIP-seq replicates and their corresponding inputs. **B:** Fingerprint plot to examine the quality of H3.3 ChIP signal in comparison with inputs. ChIP-seq is enriched as more reads are found in smaller number of bins for ChIPs compared to the input.

Figure S15

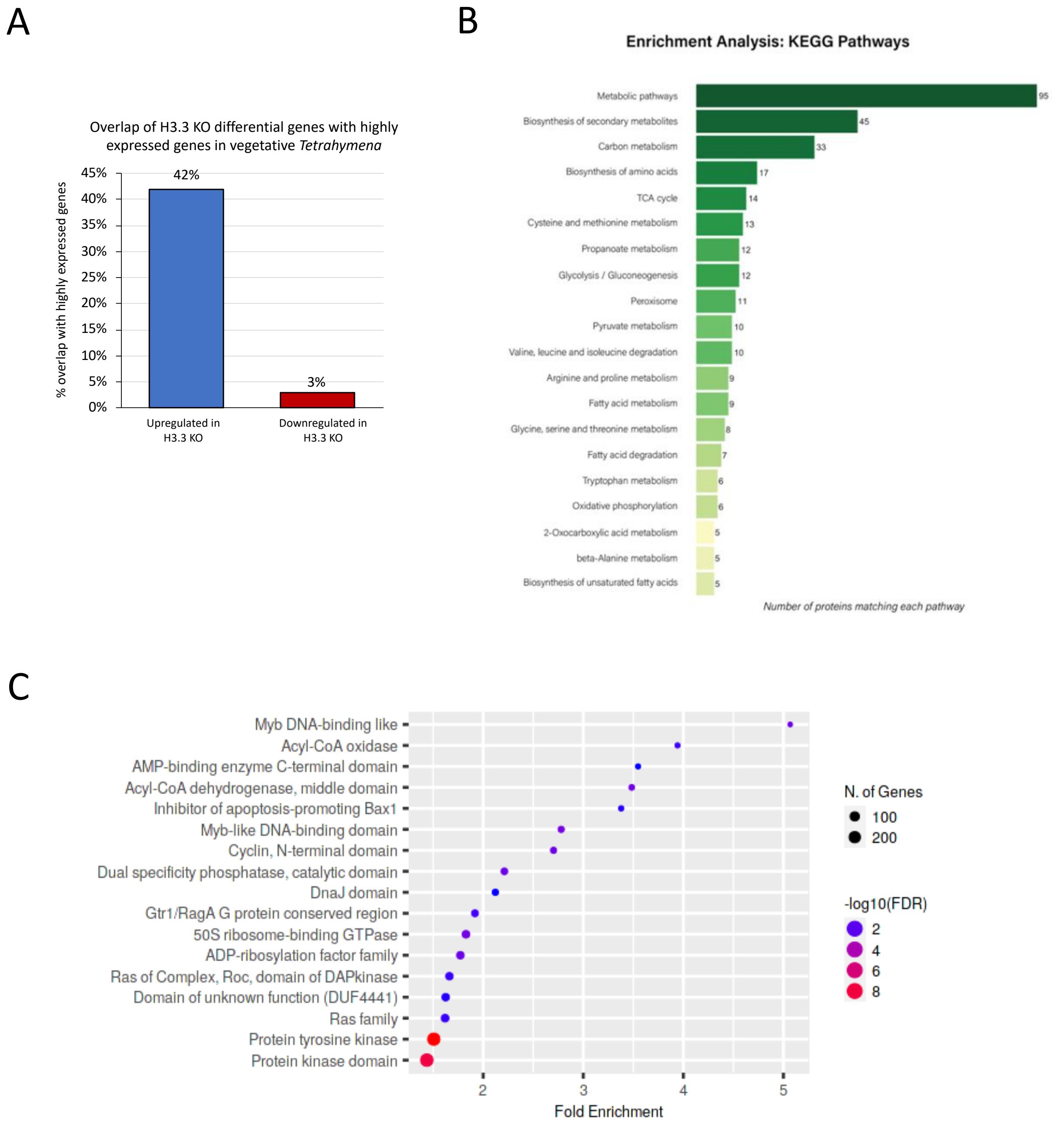

Figure S15: GO enrichment analysis of H3.3 ChIP-seq targets. A: Bar plot depicts the % overlap of upregulated and downregulated genes in H3.3 KO cells with those genes classified as highly expressed during *Tetrahymena* vegetative growth. **B:** KEGG pathway enrichment analysis of H3.3 bound genes. Number of genes for each term is indicated beside each bar. **C:** Dot plot representation of pfam domain enrichment analysis in H3.3-target genes (Q< 0.05). Figure legend is provided.

Figure S16

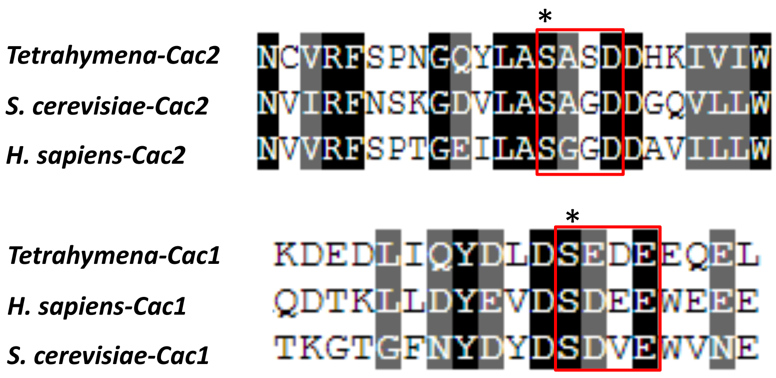

Figure S16: Predicted CKII sites on $Cac2^{Tt}$ and $Cac1^{Tt}$ proteins. The red box shows the conserved sequence whereas star indicates the serine residue predicted to be phosphorylated by CKII. The prediction was performed using Netphos web server https://services.healthtech.dtu.dk/services/NetPhos-3.1/.
