# Supplementary material for: Multilevel interrogation of H3.3 reveals a primordial role in transcription regulation
Source: Epigenetics Chromatin. 2023 Apr 7;16:10. doi: 10.1186/s13072-023-00484-9 (PMC10080907; doi:10.1186/s13072-023-00484-9)
Supplement: Supplementary file 3 — Additional file 3: Methods S1. [file 13072_2023_484_MOESM3_ESM.pdf]

Multilevel interrogation of H3.3 dynamics reveals a primordial role in transcription regulation

Syed Nabeel-Shah[1,2,3,7], Jyoti Garg [1,4,7], Kanwal Ashraf [4], Renu Jeyapala[1], Hyunmin Lee[2,5], Alexandra Petrova[1], James D Burns[2], Shuye Pu[2], Zhaolei Zhang[2,3,5], Jack F. Greenblatt[2,3], Ronald E Pearlman[4], Jean-Philippe Lambert[6] and Jeffrey Fillingham[1,*]

[1.] Department of Chemistry and Biology, Toronto Metropolitan University, 350 Victoria St., Toronto, M5B 2K3, Canada.
[2.] Donnelly Centre, University of Toronto, Toronto, M5S 3E1, Canada.
[3.] Department of Molecular Genetics, University of Toronto, Toronto, M5S 1A8, Canada.
[4.] Department of Biology, York University, 4700 Keele St., Toronto, M3J 1P3, Canada.
[5.] Department of Computer Science, University of Toronto, Toronto, M5S 1A8, Canada.
[6.] Department of Molecular Medicine, Cancer Research Center, Big Data Research Center, Université Laval, Quebec, Canada; CHU de Québec Research Center, CHUL, 2705 Laurier Boulevard, Quebec, G1V 4G2, Canada.
[7.] These authors contributed equally to this work

[*] Corresponding author: jeffrey.fillingham@torontomu.ca

Keywords: *H3.3, Histone variant, HIRA, CAF1, Asf1, NASP, RBBP4/7, Chromatin, Epigenetics, Functional proteomics, Tetrahymena*

Supplemental Methods

Generation of whole cell extracts and Western blotting

10% trichloroacetic acid (TCA) was used to prepare whole-cell extracts (WCE). The WCE was incubated on ice for 20-30 min. 100µl of SDS loading dye (1×) and 10µl of 1N NaOH was added to neutralize the solution. WCEs were electrophoresed through 10% SDS-PAGE and proteins were transferred to nitrocellulose. After blocking in 5% skim milk, the membrane was probed with the antibodies indicated. Antibodies and dilutions used were anti-Flag (1:5000; Sigma), anti-GFP (1:5000; Ab290 Abcam), anti-Actin (1:10000; Abcam).

Multiple sequence alignment and Phylogenetic analysis

Protein sequences for the *HHT2* and *HHT3* genes were obtained from the *Tetrahymena* Genome Database (www.ciliate.org) and aligned using PRALINE alignment toolbox (Simossis and Heringa 2005).

Protein sequences were retrieved using BLAST searches using NCBI non-redundant protein sequences database (https://blast.ncbi.nlm.nih.gov/Blast.cgi). Protein sequences were analyzed at the Pfam (http://pfam.sanger.ac.uk/) and SMART (http://smart.embl- heidelberg.de/) databases to examine the domain architecture (Finn et al. 2016; Letunic and Bork 2018). Protein phylogenetic analysis were carried out using the neighbour-joining method under p-distances using MEGA 7 (Kumar et al. 2016). Multiple sequence alignments were constructed using MUSCLE with default parameters. The reliability of the resulting phylogenetic trees was assessed using the bootstrap method (1000 replicas for each tree).

Chromatin Immunoprecipitation (ChIP)

ChIP-seq experiments were performed as described previously (Saettone et al. 2018; Nabeel-Shah, Garg, Saettone, et al. 2021) with following modifications. 200 mL cells were grown to a density of 2 x 10^5 cells/ mL and pelleted. Cells were washed with 200mL 10mM Tris pH7.4, resuspended in 10 mL 1X PBS and DSG was added (10mg dissolved in 200uL DMSO). Cells were pelleted and washed with 1X PBS and resuspended in 1% PFA (paraformaldehyde) in PBS. Following PFA fixation, cells were washed with cold 1X PBS three times. The chromatin was fragmented on ice by sonication with the probe sonicator, 8X8 pulses (Duty cycle: 90%, Output: Between 4 and 5) (Branson 450 Analog Sonifier).

The lysis buffer containing the sonicated chromatin was diluted 10 times using dilution buffer (50mM Tris pH 8.0, 100mM NaCl, 5mM EDTA, 2% Triton X and 0.2% deoxycholate. Chromatin immunoprecipitation was performed by incubating samples with 2µg anti-GFP antibody (ab290: Abcam) conjugated with protein G dynabeads and rotating at 4°C for 4h. The beads were washed twice with low salt wash buffer (150mM NaCl, 50mM Tris pH7.0, 0.1% SDS, 1% TritonX, 10mM EDTA, 1mM PMSF), twice with high salt wash buffer (500mM NaCl, 50mM Tris pH7.0, 0.1% SDS, 1% TritonX, 10mM EDTA, 1mM PMSF), and once with lithium wash buffer (4mM LiCl, 10mM Tris pH7.5).
Elution was performed with freshly made elution buffer (50mM Tris, 10mM EDTA, 1% SDS). Incubation was for 15min in a 65˚C water bath with brief vortexing every few minutes. 450ul of elution buffer was also added to the thawed input samples. 20ul of 5M NaCl was added and the samples were incubated overnight at 65˚C for reverse crosslinking. Samples were treated with RNase (Fermentas) and incubated for 1h at 37°C. Proteinase K (Fermentas) was added and incubated at 42°C for 2 hrs and 65°C for 8hrs. The DNA was isolated (Qiagen PCR purification kit) and H_2O was used for elution.

Mass Spectrometry sample preparation

Preparation of protein eluates for mass spectrometry acquisition was essentially as previously described (Saettone et al. 2018; Nabeel-Shah, Garg, Kougnassoukou Tchara, et al. 2021) and is provided below as it is. Briefly, the eluates were dried using a speed vacuum apparatus and re-suspended in 10µL of 20 mM Tris-HCl pH 8.0. Trypsin digestion was carried out using 0.75 µg of trypsin (Sigma) for ~ 15 hours at 37°C with mild agitation. An extra 0.25 µg of trypsin was added to each sample and incubated for an additional 3 h. The samples were acidified to a final concentration of 2% acetic acid, desalted using C_{18}StageTips (Thermo Scientific) as per the manufacturer's instructions and stored at -80°C until their acquisition on a mass spectrometer.

Mass spectrometry acquisition using Triple TOF 5600 mass spectrometer

5 µL of each sample, representing 50% of the sample, was directly loaded at 300 nL/min onto a New Objective PicoFrit column (15 cm×0.075 mm I.D; Scientific Instrument Services, Ringoes, NJ) packed with Jupiter 5 µm C_{18} (Phenomenex, Torrance, CA) stationary phase. The peptides were eluted from the column by a gradient generated by an Agilent 1200 HPLC system (Agilent,

Santa Clara, CA) equipped with a nano electrospray ion source coupled to a 5600+ Triple TOF mass spectrometer (Sciex, Concord, ON). A 65-min linear gradient of a 5–35% mixture of 0.1% formic acid injected at 300 nL/min was used to elute peptides. Data dependent acquisition mode was used in Analyst version 1.7 (Sciex) to acquire mass spectra. Full scan mass spectrum (400 to 1250m/z) were acquired followed by collision-induced dissociation of the twenty most intense ions. A period of 20 s and a tolerance of 100 ppm were set for dynamic exclusion.

Mass spectrometry acquisition using Orbitrap Fusion mass spectrometer

Peptide samples were separated by online reversed-phase (RP) nanoscale capillary liquid chromatography (nanoLC) and analyzed by electrospray mass spectrometry (ESI MS/MS). The experiments were performed with a Dionex UltiMate 3000 nanoRSLC chromatography system (Thermo Fisher Scientific) connected to an Orbitrap Fusion mass spectrometer (Thermo Fisher Scientific) equipped with a nanoelectrospray ion source. Peptides were trapped at 20 ul / min in loading solvent (2% acetonitrile, 0.05% TFA) on a 5mm x 300 µm C18 pepmap cartridge pre-column (Thermo Fisher Scientific) during 5 minutes. The pre-column was switched online with a self-made 50 cm x 75 µm internal diameter separation column packed with ReproSil-Pur C18-AQ 3-µm resin (Dr. Maisch HPLC) and the peptides were eluted with a linear gradient from 5-40% solvent B (A: 0,1% formic acid, B: 80% acetonitrile, 0.1% formic acid) in 60 minutes, at 300 nL/min. Mass spectra were acquired using a data dependent acquisition mode using Thermo XCalibur software version 3.0.63. Full scan mass spectra (350 to 1800m/z) were acquired in the orbitrap using an AGC target of 4e5, a maximum injection time of 50 ms and a resolution of 120 000. Internal calibration using lock mass on the m/z 445.12003 siloxane ion was used. Each MS scan was followed by acquisition of fragmentation spectra of the most intense ions for a total cycle time of 3 seconds (top speed mode). The selected ions were isolated using the quadrupole analyzer in a window of 1.6 m/z and fragmented by Higher energy Collision-induced Dissociation (HCD) with 35% of collision energy. The resulting fragments were detected by the linear ion trap in rapid scan rate with an AGC target of 1E4 and a maximum injection time of 50ms. Dynamic exclusion of previously fragmented peptides was set for a period of 20 sec and a tolerance of 10 ppm.

Mass spectrometry acquisition using LTQ mass spectrometer

The suspended sample was bomb-loaded in its entirety on the equilibrated column. The column was washed off-line for 10 min in buffer A and placed in-line with a LTQ mass spectrometer equipped with an Agilent 1100 pump with split flow, and either the Thermo source, or a Proxeon source. Buffer A is 2% acetonitrile (ACN), 0.1% formic acid; buffer B is 98% ACN, 0.1% formic acid. The HPLC gradient program delivered an ACN gradient over 120 min (1-5% buffer B over 4 min, 5-40% buffer B over 100 min, 40-60% buffer B over 5 min, 60-100% buffer B over 5 min, hold buffer B at 100% 3 min, and 100-0%B in 2 min). The parameters for Data Dependent Acquisition on the mass spectrometer were: 1 centroid MS (mass range 400-2000) followed by MS/MS on the 5 most abundant ions. General parameters were activation type = CID, isolation width = 3, normalized collision energy = 32, activation Q = 0.25, activation time = 30 msec, wide band activation. The minimum threshold was 1000, the repeat4count = 1, repeat duration = 30 sec, exclusion size list = 500, exclusion duration = 30sec, exclusion mass width (by mass) = low 1.2, high 1.5.

Data Dependent Acquisition MS analysis:

Mass spectrometry data were stored, searched and analyzed using the ProHits laboratory information management system (LIMS) platform (Liu et al. 2016). Within ProHits, Thermo Fisher scientific RAW mass spectrometry files were converted to mzML and mzXML using ProteoWizard (3.0.4468; (Kessner et al. 2008). Within ProHits, AB SCIEXWIFF files were first converted to an MGF formatusing WIFF2MGF converter and to an mzML format using ProteoWizard (v3.0.4468) and the AB SCIEX MS Data Converter (V1.3 beta). The mzML and mzXML files were searched using Mascot (v2.3.02). The spectra were searched with the RefSeq database (version45, January 24th, 2011) acquired from NCBI against a total of 24,770 *T. thermophila* sequences. For TripleTOF files, the database parameters were set to search for tryptic cleavages, allowing up to two missed cleavage sites per peptide with a mass tolerance of 40 ppm for precursors with charges of 2+ to 4+ and a tolerance of +/- 0.15 amu for fragment ions. For Orbitrap Fusion files, the database parameters were set to search for tryptic cleavages, allowing up to two missed cleavage sites per peptide with a mass tolerance of 12 ppm for precursors with charges of 2+ to 4+ and a tolerance of +/- 0.6 amu for fragment ions. For files analyzed on the LTQ, the charges +1, +2 and+3 were considered, with the parent mass tolerance set at 3 amu and the fragments at 0.6 amu. Deamidated asparagine and glutamine and oxidized methionine were

allowed as variable modifications. SAINTexpress version 3.61 (Teo et al. 2014) was used as a statistical tool to calculate the probability value of each potential protein-protein interaction from background contaminants using default parameters. For the LTQ samples, 39 controls compressed to 20 were used while 15 uncompressed controls were employed for the Triple TOF samples.

Supplementary References

Finn RD, Coggill P, Eberhardt RY, Eddy SR, Mistry J, Mitchell AL, Potter SC, Punta M, Qureshi M, Sangrador-Vegas A, et al. 2016. The Pfam protein families database: towards a more sustainable future. *Nucleic Acids Res.* 44:D279–D285.

Kessner D, Chambers M, Burke R, Agus D, Mallick P. 2008. ProteoWizard: open source software for rapid proteomics tools development. *Bioinformatics* 24:2534–2536.

Kumar S, Stecher G, Tamura K. 2016. MEGA7: Molecular Evolutionary Genetics Analysis Version 7.0 for Bigger Datasets. *Mol. Biol. Evol.* 33:1870–1874.

Letunic I, Bork P. 2018. 20 years of the SMART protein domain annotation resource. *Nucleic Acids Res.* 46:D493–D496.

Liu G, Knight JDR, Zhang JP, Tsou C-C, Wang J, Lambert J-P, Larsen B, Tyers M, Raught B, Bandeira N, et al. 2016. Data Independent Acquisition analysis in ProHits 4.0. *J. Proteomics* 149:64–68.

Nabeel-Shah S, Garg J, Kougnassoukou Tchara PE, Pearlman RE, Lambert JP, Fillingham J. 2021. Functional proteomics protocol for the identification of interaction partners in Tetrahymena thermophila. *STAR Protoc.* 2.

Nabeel-Shah S, Garg J, Saettone A, Ashraf K, Lee H, Wahab S, Ahmed N, Fine J, Derynck J, Pu S, et al. 2021. Functional characterization of RebL1 highlights the evolutionary conservation of oncogenic activities of the RBBP4/7 orthologue in Tetrahymena thermophila. *Nucleic Acids Res.* 49:6196–6212.

Saettone A, Garg J, Lambert J-P, Nabeel-Shah S, Ponce M, Burtch A, Thuppu Mudalige C, Gingras A-C, Pearlman RE, Fillingham J. 2018. The bromodomain-containing protein Ibd1 links multiple chromatin-related protein complexes to highly expressed genes in Tetrahymena thermophila. *Epigenetics Chromatin* 11:10.

Simossis VA, Heringa J. 2005. PRALINE: a multiple sequence alignment toolbox that integrates homology-extended and secondary structure information. *Nucleic Acids Res.* 33:W289.

Teo G, Liu G, Zhang J, Nesvizhskii AI, Gingras A-C, Choi H. 2014. SAINTexpress:

improvements and additional features in Significance Analysis of INTeractome software. *J. Proteomics* 100:37–43.
